# Revelation of Interfacial Energetics in Organic Multiheterojunctions

**DOI:** 10.1002/advs.201600331

**Published:** 2016-12-01

**Authors:** Christian Kästner, Koen Vandewal, Daniel Ayuk Mbi Egbe, Harald Hoppe

**Affiliations:** ^1^ Institute of PhysicsTechnische Universität IlmenauWeimarer Str. 3298693IlmenauGermany; ^2^ Institut für Angewandte PhotophysikTechnische Universität DresdenGeorge‐Bähr‐Str. 101069DresdenGermany; ^3^ Institute of Polymeric Materials and TestingJohannes Kepler University LinzAltenbergerstr. 694040LinzAustria

**Keywords:** bulk heterojunction, charge transfer state, electroluminescence, interfacial order, photoluminescence, polymer:fullerene solar cells

## Abstract

Efficient charge generation via exciton dissociation in organic bulk heterojunctions necessitates donor–acceptor interfaces, e.g., between a conjugated polymer and a fullerene derivative. Furthermore, aggregation and corresponding structural order of polymer and fullerene domains result in energetic relaxations of molecular energy levels toward smaller energy gaps as compared to the situation for amorphous phases existing in homogeneously intermixed polymer:fullerene blends. Here it is shown that these molecular energy level shifts are reflected in interfacial charge transfer (CT) transitions and depending on the existence of disordered or ordered interfacial domains. It can be done so by systematically controlling the order at the donor–acceptor interface via ternary blending of semicrystalline and amorphous model polymers with a fullerene acceptor. These variations in interfacial domain order are probed with luminescence spectroscopy, yielding various transition energies due to activation of different recombination channels at the interface. Finally, it is shown that via this analysis the energy landscape at the organic heterojunction interface can be obtained.

## 1 Introduction

Photon absorption in organic semiconductors generally leads to excitons, i.e., bound electron–hole pairs, exhibiting a binding energy in the range of 0.1–1 eV.^1^ This is in contrast to inorganic semiconductors, where exciton binding energies are below the thermal energy (*k*_B_ *T*), leading to free charge carriers at room temperature upon photon absorption as in silicon solar cells.^2^ These high exciton binding energies in organic semiconductors can be largely assigned to be due to comparatively small dielectric constants lately leading to a quest for obtaining high‐permittivity organic semiconductors by intelligent material design.^3^, ^4^, ^5^

As the low dielectric constant limits charge generation in organic semiconductors, organic solar cells require an additional process for exciton dissociation into free charge carriers, which is conveniently realized at an interface between two different molecules exhibiting an energy level offset in the range of or larger than the exciton binding energy of the constituting organic semiconductors. The semiconductor exhibiting higher molecular energy levels—specifically the lowest unoccupied molecular orbital (LUMO) but also the highest occupied molecular orbital (HOMO)—is then called the (electron) donor, whereas the other is called the (electron) acceptor. This so‐called type II heterojunction enables efficient electron transfer from the donor to the acceptor, which may be accompanied by the formation of charge transfer (CT) states, where the electron is located on the acceptor LUMO and the hole on the donor HOMO. In order to maximize the charge generation yield in organic solar cells considering limited exciton diffusion lengths in both materials, the so‐called bulk heterojunction concept has been introduced, which enables charge generation throughout the bulk of the blend film by intimately mixing of donor and acceptor phases at the nanoscale.^6^, ^7^, ^8^

Since then intense research efforts were conducted to investigate, modify, and ultimately control the phase separation between the commonly used polymer donors and fullerene‐based acceptors. Since too intimate mixtures do not only promote charge generation but also increase charge recombination rates, balancing the domain sizes constituting the bulk heterojunction is part of the optimization process for maximizing photovoltaic device efficiencies.^7^, ^9^, ^10^, ^11^, ^12^, ^13^, ^14^, ^15^, ^16^, ^17^, ^18^ For controlling domain sizes, various processing parameters have been tuned: among them choice of solvents,^17^, ^19^, ^20^ use of nonsolvents promoting aggregation of polymers,^20^, ^21^, ^22^ post‐production thermal^23^, ^24^ or solvent^25^, ^26^ annealing as well as use of processing additives selectively dissolving one of the two organic components and causing its aggregation within the blend film during drying.^27^, ^28^

In general it has been demonstrated that the aggregation of both materials may improve charge generation, separation, and transport.^11^, ^29^, ^30^, ^31^, ^32^, ^33^, ^34^, ^35^, ^36^ Whilst exciton dissociation and recombination rates are proportional to the donor–acceptor interfacial area,^35^, ^37^ charge transport is governed by domain size, percolation, purity, and structural order.^7^, ^14^, ^16^, ^38^ One major important point is that semiconductors—regardless of whether they are organic or inorganic—yield smaller energy gaps (or respectively reduced HOMO–LUMO energy separation), when ordering occurs.^39^, ^40^ This effect is based on an increased energetic overlap due to the higher volume density of electronic interactions, respectively, wave functions in the system, caused by spatially more close and energetically more equivalent electronic states. Thus to date there is an ongoing quest for the reliable determination of such energetic effects due to differences in order or crystallinity of organic semiconductors.^41^

Besides the above mentioned two‐step charge generation process, constituted of an excitation of the donor material followed by a charge transfer to the acceptor or vice versa, lately a single step charge generation process via direct excitation of the CT‐state located at the donor–acceptor interface has been discovered and discussed with growing intensity.^42^, ^43^, ^44^, ^45^, ^46^, ^47^ Early studies using highly sensitive external quantum efficiency measurements already revealed that below both the electronic transitions of neat donor and acceptor in the blend an additional very weak transition exists, representing the CT.^42^, ^43^, ^44^, ^45^, ^46^, ^48^, ^49^, ^50^, ^51^, ^52^, ^53^, ^54^ Furthermore it has been shown by electroluminescence (EL) measurements that also the opposite process, i.e., luminescent recombination via a CT‐state, can be detected.^42^, ^43^, ^44^, ^45^, ^54^, ^55^ One major conclusion was that the transition energy of this CT‐state is inherently linked to the energetic separation of charge carriers and thus to the open‐circuit voltage of organic solar cells.^43^, ^56^, ^57^ This observation led to the discussion whether the CT‐state is always an intermediate step within the charge generation process.^47^, ^58^ On the other hand, time‐resolved measurements have shown that roughly 1/3rd of all charge generation processes proceed over the two‐step process, whereas the majority (2/3rd) of charge carriers is more or less instantaneously generated without the intermediate step of exciton formation within either donor or acceptor.^59^, ^60^ In summary, both excitation processes do generally coexist within organic bulk heterojunctions and they often have the same quantum efficiency for charge generation.^45^

**Figure 1:**
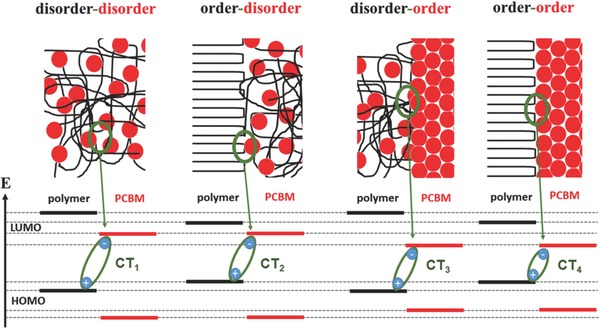
Energy level alignment for different possible interfaces hypothetically formed in donor–acceptor bulk heterojunctions containing both, ordered and disordered phases of both, donor and acceptor, emphasizing the resulting variations in charge transfer state energy. In this case, without limitation of generality, a polymer constitutes the donor, whereas a fullerene derivative represents the acceptor. More ordered phases at the heterojunction yield lower transition energies of the intermolecular charge transfer state.

We hypothesize that the energetic position of the CT transition of donor–acceptor bulk heterojunctions depends directly on the interfacial order of the two phase domains and can thus be probed by a combination of photoluminescence (PL)^52^, ^55^, ^61^, ^62^ and electroluminescence spectroscopy.^43^, ^61^, ^63^, ^64^ This hypothesis is illustrated schematically in **Figure** **1** for the case of organic donor–acceptor heterojunctions, in which both of the constituting materials are able to form either ordered (crystalline) or disordered (amorphous) phases. Thus interfacing the donor phase (here, polymer) with the acceptor phase (here, fullerene derivative) can potentially yield four structurally different interfaces: disorder–disorder, order–disorder, disorder–order, and order–order. However, the corresponding four CT‐transitions have not been experimentally observed so far within one single bulk heterojunction. It was recently shown that structural order and crystallinity improve the charge separation due to energetic relaxation within ordered phases.^64^, ^65^, ^66^, ^67^ Hence aggregation‐induced energetic shifts of the donor HOMO and the acceptor LUMO will lead to changes in CT‐transition energies. Thus, luminescence spectroscopy in CT emission region should enable us to measure the structural order at the donor–acceptor interface thereby providing deeper insight into the fine‐scale morphology of bulk heterojunctions.

One option to modify the photovoltaic parameters and the morphology of bulk heterojunction solar cells distinctly, is ternary blending of three components, either two polymers and one fullerene or one polymer and two fullerenes.^31^, ^68^, ^69^, ^70^, ^71^ In the presented study ternary blends of semicrystalline AnE‐PV*ab* (anthracene‐containing poly(p‐phenylene‐ethynylene)‐alt‐poly(p‐phenylene‐vinylene) (PPE‐PPV) copolymers (AnE‐PV), equipped with octyloxy side‐chains at the PPE‐part and 2‐ethylhexyloxy side‐chains at the PPV‐part) and amorphous AnE‐PV*ba* (AnE‐PV equipped with 2‐ethylhexyloxy side‐chains at the PPE‐part and octyloxy side‐chains at the PPV‐part)—both structures may be found in the Supporting Information—in various ratios were applied to create those different interfaces as mentioned above.^33^, ^34^, ^72^, ^73^ Thereby, for simplicity and comparability, the overall polymer:PCBM (phenyl‐C61‐butyric acid methyl ester) ratio was held constant. Due to the chemical and physical properties of both polymer analogues, the bulk morphology could indeed be precisely tuned between mostly phase‐separated and ordered to homogenously intermixed and completely disordered.^33^, ^34^, ^72^, ^73^ Consequently, the interfaces between the, in composition and structural order varying, phase domains were probed with photoluminescence and electroluminescence spectroscopy. Both measurement methods revealed different spectral responses in the observed CT region due to the difference in the underlying exciton and/or charge carrier recombination pathway. Ultimately, it is demonstrated via luminescence spectroscopy that various different CT emission peaks within one single bulk heterojunction exist and that these peaks indeed yield direct information about the interfacial order of donor and acceptor phases. Based on these CT transitions, we were furthermore able to reveal to the best of our knowledge for the first time the energy difference between amorphous and crystalline phases in organic donor–acceptor bulk heterojunctions spectroscopically.

## 2 Results and Discussion

**Figure 2:**
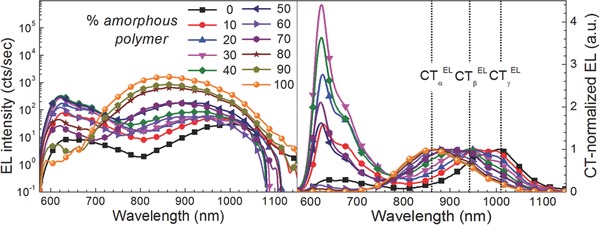
Electroluminescence spectra for varying concentrations of amorphous polymer (AnE‐PV*ba*) in ternary (AnE‐PV*ab*:AnE‐PV*ba*:PCBM) blends; left: as measured and right: normalized to the dominant charge transfer transition.

In order to address the potential recombination channels depicted in Figure 1, electroluminescence spectra were recorded on complete solar cells.^33^ The results of these measurements are depicted in **Figure** **2** in two representations: as obtained under constant current density of 200 mA cm^−2^ (left) and normalized to the CT‐emission peaks (right). From the left graph in Figure 2 it is obvious that the relative intensity of the different CT peaks is strongly varying over two orders of magnitude, emphasizing the necessity of highly sensitive detection setups having a low noise level. To the naked eye three different CT transitions with varying oscillator strength, depending on the composition ratio of semicrystalline and amorphous polymer, are visible in the right graph in Figure 2 and represent a novelty. A first transition is seen at around 859 nm (${\mathrm{CT}}_{\alpha }^{\mathrm{EL}}$), a second transition at around 942 nm (${\mathrm{CT}}_{\beta }^{\mathrm{EL}}$) and a third transition at around 1007 nm (${\mathrm{CT}}_{\gamma }^{\mathrm{EL}}$). Even though already three CT transitions are clearly visible, this observation seems to be in contradiction to our expectation of four different recombination channels, as depicted in Figure 1. In order to gain higher confidence, the respective CT emission spectra were subdued to a quantitative analysis of fitting them by either Gaussian, Lorentzian, or their convolutions, Voigt profiles.

According to the literature, CT absorption bands can usually be fitted by Gaussian functions.^44^, ^74^, ^75^, ^76^ However, in our case, only one could be sufficiently well fitted by a single, pure Gaussian line shape function. All other peaks could not be satisfactorily fitted. Since pure Lorentzians did not fit either, Voigt profiles, i.e., convolutions of Gaussians and Lorentzians,^77^, ^78^ were chosen. Physically seen, the Gaussian width within the Voigt profile could be a measure for the disorder‐induced inhomogeneous broadening,^76^ whereas the Lorentzian width is the measure for the lifetime‐dependent homogeneous broadening. In principle the Voigt profile is also only an approximation to more sophisticated line shape functions based on dissipative quantum theory on various time scales.^76^

Intriguingly, yet working for some of the compositions with lower contents of the amorphous polymer fraction, a set of three different Voigt profiles did not yield satisfactory results for all blends investigated. By applying one more Voigt profile, i.e., allowing an additional emission peak, to the fitting procedure, however, good fits could be simultaneously obtained for all blend ratios between the semicrystalline and the amorphous polymer. For detailed information about the individual fit results the reader is referred to the Supporting Information. Due to possible reorganization, the electronic CT‐state energy is often given as an intersection between the electroluminescence and the external quantum efficiency spectra of a given solar cell.^42^, ^44^, ^76^ However, this correlation holds only for the overdamped multimode Brownian oscillator model in the high‐temperature limit,^79^ which should result in neat Gaussian‐shaped peaks. Due to the fact that our fits require Voigt profiles, having considerable contributions from Lorentzian line shape functions, it is obvious that the assumptions of this model do not hold here. Therefore, the CT‐energies were taken from the center peak positions without modification, which corresponds to the lowest accessible CT‐state in absorption according to Vandewal et al.^47^

**Figure 3:**
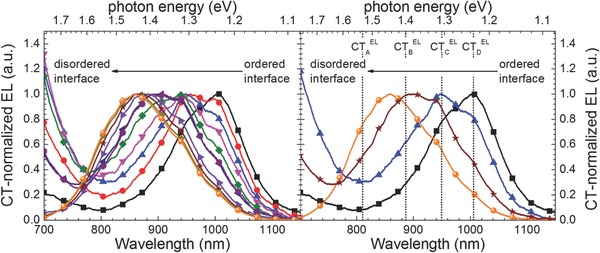
CT transition peaks obtained from the fitting procedure by application of Voigt profiles yielding four different specific CT peaks, centred around CT_A_^EL^ ≈ 1.51 eV, CT_B_^EL^ ≈ 1.39 eV, CT_C_^EL^ ≈ 1.31 eV, and CT_D_^EL^ ≈ 1.23 eV.

**Figure 4:**
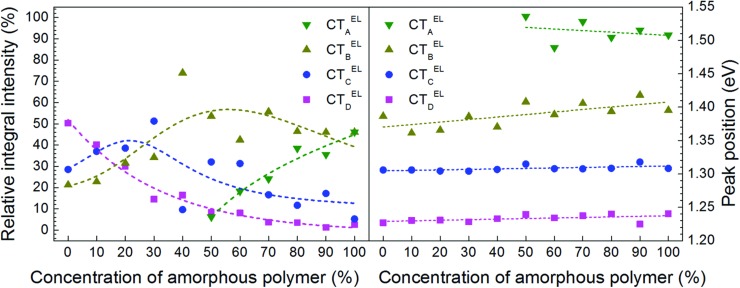
Relative integrated electroluminescence intensities of the detected charge transfer transitions as a function of the amorphous polymer concentration (broken lines are guide to the eye) (left). Voigt profile peak positions in CT‐electroluminescence spectra in dependence of the concentration of amorphous polymer (right).

Roughly, the from electroluminescence obtained CT peak positions consistent for all blend compositions are around CT_A_^EL^ ≈ 1.51 eV, CT_B_^EL^ ≈ 1.39 eV, CT_C_^EL^ ≈ 1.31 eV, and CT_D_^EL^ ≈ 1.23 eV. These peak positions are depicted in **Figure** **3** right. Nevertheless, small peak shifts for different blend compositions (see also **Figure** **4** right) were inevitable, which seem to be unphysical at first glance. After some consideration, however, it may be plausible that with increasing amorphous polymer fractions, an accordingly changing dielectric environment of the emitter may in return cause shifting the position of the dipolar CT‐transition.^80^

After having obtained the expected number of CT transitions and their spectral positions, their assignment to individual heterojunction interfaces is still required. Since aggregation into semicrystalline regions leads to a lower energy gap, the two extremal peak positions could be simply assigned: following the hypothesis depicted in Figure 1 the highest energy peak CT^EL^_A_ corresponds to the amorphous/amorphous or disorder–disorder heterojunction CT_1_^EL^ (CT_1_ in Figure 1), whereas the peak lowest in energy (CT_D_^EL^) relates to the semicrystalline/semicrystalline or order–order heterojunction (CT_4_^EL^). In order to assign the peaks with intermediate energies (CT_B_^EL^ and CT_C_^EL^), their occurrence with respect to the relative amount of semicrystalline polymer or PCBM domains is crucial. For that the directly from the fitting procedure resulting integrated normalized relative electroluminescence contributions of the CT transitions were analyzed, as shown in Figure 4 left. Since the CT_2_^EL^ transition is only probable under presence of semicrystalline polymer,^33^ the lower in energy CT_C_^EL^ peak, significantly present in blend ratios containing less than 50% of the amorphous polymer (AnE‐PV*ba*), corresponds to it. Thus the remaining CT_B_^EL^ transition can be assigned to the transition between aggregated PCBM and amorphous polymer (AnE‐PV*ba*), i.e., CT_3_^EL^. It is interesting to mention that the CT_A_^EL^ = CT_1_^EL^ transition, taking place for intimately mixed PCBM molecules with amorphous polymer in the expected range of high amount of amorphous polymer (see Figure 4), nicely corresponds to the occurrence of a kind of intercalated phase, as detected by GiWAXS measurements earlier.^33^

Quantitatively, the CT peak energies and their trend on increasing amorphous polymer concentration could be evaluated by a linear fit as shown in Figure 4 right. The extracted values for peak mean energies and their shift with concentration of the amorphous polymer fraction are depicted in **Table** **1**.

**Table 1:** Fit values of Voigt peaks extracted from data shown in Figure 4 right

Δ*E*		Peak mean‐value [eV]	Peak shift gradient [meV/%]	Maximum deviation [eV]
CT_A_^EL^	CT_1_^EL^	1.513	−0.241	±0.032
CT_B_^EL^	CT_3_^EL^	1.386	0.376	±0.017
CT_C_^EL^	CT_2_^EL^	1.308	0.072	±0.010
CT_D_^EL^	CT_4_^EL^	1.233	0.084	±0.008

The results of electroluminescence spectroscopy indeed confirm the hypothesis for the existence of four different CT‐state recombination channels at differently ordered domain interfaces. In addition, percolating pristine semicrystalline polymer phases yield considerable amount of light emission, which has to be assigned to lateral phase separation within the blend film as detected by laterally resolved electroluminescence imaging described elsewhere.^73^, ^81^

**Figure 5:**
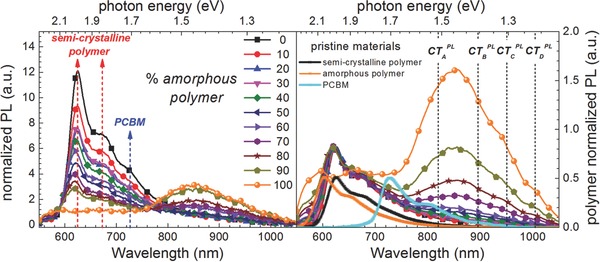
Photoluminescence spectra for various concentrations of amorphous polymer (AnE‐PV*ba*) in ternary blends; left: normalized to thin film absorption at excitation wavelength and right: normalized to the polymer emission.

**Figure 6:**
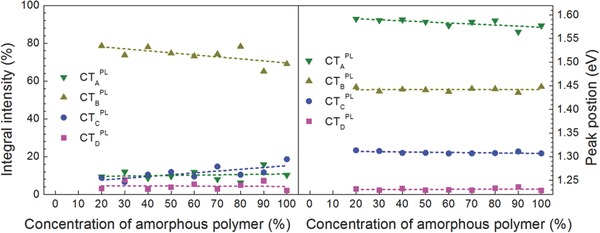
Relative integrated photoluminescence intensities of the detected charge transfer transitions as a function of the amorphous polymer concentration (broken lines are linear fits as guide to the eye) (left). Voigt profile peak positions in CT‐photoluminescence spectra in dependence of the concentration of amorphous polymer (right).

Next, the PL spectra, which correspond to probing the bulk volume properties, were analyzed. **Figure** **5** left shows the PL spectra normalized to the absorption at the excitation wavelength of the laser and Figure 5 right normalized to the polymer emission. Intriguingly, and in contrast to the electroluminescence data, no strong variation in the distribution of CT‐transition energies can be found. It is obvious, that besides CT‐transitions, also the neat polymer phase, specifically the semicrystalline polymer representative, and also the neat PCBM phase yield PL emission. The latter emissions have to be assigned to excitations within large enough domains that do not yield charge separation at the heterojunction interface, but rather lead to radiative recombination in a single material phase.^35^ To further analyze the involved CT‐transitions, the lower in energy spectral range was again subdued to a fitting procedure involving Voigt profiles (see Supporting Information). The results of this analysis are depicted in **Figure** **6**. Note that for very low fractions of the amorphous polymer insufficient signal strength prevented obtaining any fit results, and these data points are consequently left out. The CT peak energies and their trend on increasing amorphous polymer fraction concentration could be again evaluated by a linear fit as shown in figure 6 right. The extracted values for peak mean energies and their shift with concentration of the amorphous polymer fraction are depicted in **Table** **2**.

**Table 2:** Fit values of Voigt peaks extracted from data shown in Figure 6 right

Δ*E*		Peak mean‐value [eV]	Peak shift gradient [meV/%]	Maximum deviation [eV]
CT_A_^PL^	CT_1_^PL^	1.583	−0.022	±0.019
CT_B_^PL^	CT_3_^PL^	1.442	0.000	±0.006
CT_C_^PL^	CT_2_^PL^	1.309	−0.005	±0.004
CT_D_^PL^	CT_4_^PL^	1.231	0.001	±0.005

In contrast to the electroluminescence study, no strong variations in peak strength with respect to the concentration of the amorphous polymer can be detected, but as clearly seen in Figure 5, the photoluminescence is dominated by a single CT‐recombination energy. Furthermore, as can be seen from the Supporting Information the overall signal strength increases for larger amorphous polymer fractions. It should be pointed out here, that photoluminescence is probing the bulk, i.e., the volume properties of the corresponding donor–acceptor mixtures, whereas in case of electroluminescence those interfaces are highlighted, that can be reached via charge percolation from the electrodes. Indeed, there are some specific differences observed with respect to the CT‐transition energies: whereas CT_C_^PL^ and CT_D_^PL^ fully correspond energetically to those found in electroluminescence spectra, CT_A_^PL^ and CT_B_^PL^ differ significantly from those in the electroluminescence. The average CT‐transition energies observed via PL are thus: CT_A_^PL^ ≈ 1.58 eV, CT_B_^PL^ ≈ 1.44 eV, CT_C_^PL^ ≈ 1.31 eV, and CT_D_^PL^ ≈ 1.23 eV. Due to the good agreement of the CT_C_ and CT_D_ peaks for EL and PL, the overall assignment to the corresponding molecular interfaces must be the same as for electroluminescence.

The difference in transition energies between CT_1_^PL^/CT_3_^PL^ as compared to CT_1_^EL^/CT_3_^EL^ has to be assigned physically to a different transition at the heterojunction interface: according to Sweetnam et al. there exists a difference in the HOMO energy of neat amorphous polymer and amorphous polymer intermixed (or even intercalated) with PCBM.^41^ Sweetnam et al. experimentally detected by using cyclic voltammetry that the amorphous polymer intermixed with the fullerene yields a higher spread in energy levels, leading to a deeper HOMO level. In agreement with this finding we assign the CT_1_^PL^ to a transition between the amorphous PCBM and the amorphous polymer molecularly intermixed with PCBM (intercalated phase),^33^ whereas the CT_3_^EL^ transition corresponds to a transition from aggregated PCBM to the polymer in the intercalated phase. We conclude that the dominance of the CT_3_^EL^ peak correlates well with the fact that most of the volume inside the donor–acceptor mixture consists of amorphous polymer intercalated by PCBM adjacent to PCBM aggregates. Note that in GiWAXS spectra, obtained on the same blends earlier, indeed the occurrence of a broad peak was detected for higher amorphous polymers fractions, which had been assigned to a specific molecularly intermixed, intercalated polymer:fullerene phase.^33^

To further reason that in electroluminescence the recombination takes place between the neat amorphous polymer phase and either isolated or aggregated PCBM, we argue that charge injection from the electrodes as well as charge transport will be much more efficient within the neat instead the intercalated amorphous polymer. Furthermore, due to the energy offset, it will be highly improbable that holes transported within the neat amorphous polymer will overcome the energy barrier to access the intercalated amorphous polymer, whereas holes transported within the intercalated phase most probably will relax to the neat amorphous polymer phase. It has been recently shown by Gelinas et al. that charge generation can be promoted over relatively large separations, i.e., upon charge transfer, electron and hole can be separated by a few nanometers directly and with no measureable time delay into delocalized charge‐separated (CS) states within crystalline domains.^66^ The charge transfer yielding spatially separated charge carriers could be modeled for the two cases of (a) tunneling (following Fermi's Golden Rule) and (b) injection of a fully coherent electron wave packet.^66^ On the other hand, it can be comprehended that in the opposite process, taking place during charge flooding in electroluminescence characterizations, charge carriers will be occupying the same delocalized CS states and may recombine, using the same routes, over fairly large distances as well. Furthermore, it is generally well‐known and was also explicitly shown for this material system,^33^, ^34^ that the quantum efficiency of charge generation is higher for more crystalline polymer domains. Hence, the effects described here above should also apply for our material system.

As a consequence, specifically for the CT_4_ transition involving the two semicrystalline phases of polymer and fullerene, which could be separated in space due to the inevitable existence of an amorphous polymer halo around any polymer crystallite,^82^ we assume an equivalence or a negligible difference between the CT_4_ transition energy and the energetic separation of free and delocalized charge carriers in this case. In other words, we consider the CT_4_ transition to occur directly via tunneling or resonant coupling between the delocalized CS states in the semicrystalline regions. As a consequence, the CT_4_ transition should not include lowering of the energy separation between electron and hole due to Coulomb relaxation.

In principle, the remaining three CT‐transition energies are not identical but correlated with the transport levels of free charge carriers. In order to yield the CS energy, in general an individual energy offset of Δ*E* is to be added to any CT‐transition energy.^83^ As these energy level offsets are presently unknown, we may now only derive a lower limit for the energy splitting due to disorder–order effects, if we consider the differences between the evaluated CT‐transition energies that have one energy level in common, disregarding the CT_4_ transition. For example, the PCBM energy level split between the amorphous and the crystalline phase may be derived by subtraction of CT_1_^PL/EL^ − CT_3_^PL/EL^ yielding on average about 134 meV. In the same way the energy split between neat amorphous polymer and PCBM intercalated amorphous polymer with respect to the crystalline polymer phase yield 205 and 274 meV. These values serve as a lower limit, as any additional effects due to reorganization energy or Coulomb binding energy with respect to free charge carriers within the molecular energy levels are being neglected so far. Since both, the reorganization energy as well as the Coulomb binding energy should yield larger effects, the higher the disorder, we may indeed take the differences from CT‐transition energies as lower limit for the energy split due to disorder effects.

The resulting lower limit for energy level offsets Δ*E*_a‐c_ between the amorphous HOMO/LUMO and the crystalline HOMO/LUMO levels are summarized in **Table** **3**.

**Table 3:** Summary of obtained energy levels

Energy level	Energy offset Δ*E*_a‐c_
LUMO PCBM amorphous	
LUMO PCBM semicrystalline	>(134 ± 41) meV
HOMO polymer semicrystalline	
HOMO polymer amorphous (neat, α)	>(205 ± 34) meV
HOMO polymer amorphous (mixture, *αβ*)	>(274 ± 21) meV

**Figure 7:**
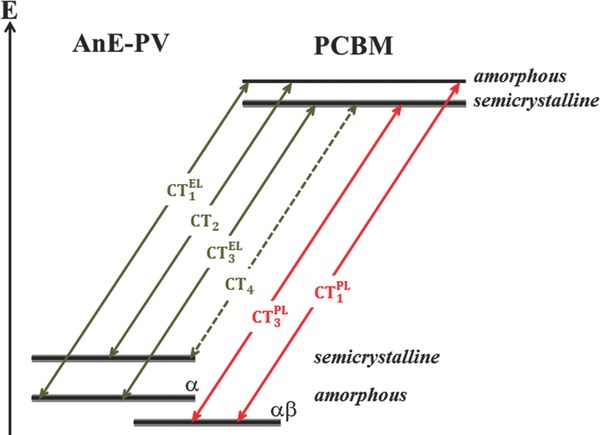
Reconstruction of interfacial energy diagram of the organic multiheterojunction.

This reconstruction of the interfacial energy diagram of the organic multiheterojunction is depicted in **Figure** **7** (for details of the calculation see the Supporting Information).

**Figure 8:**
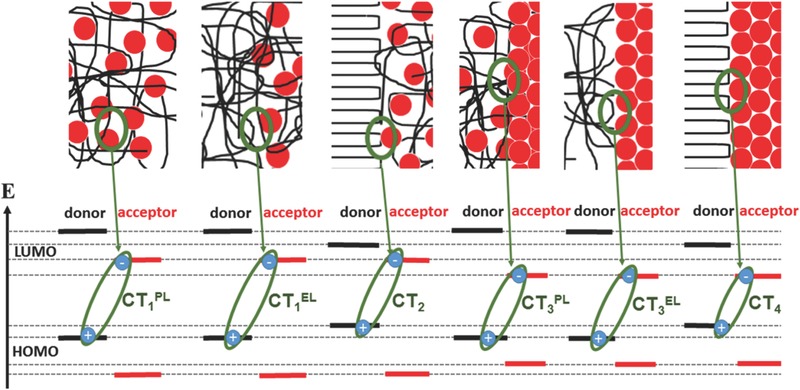
Real space sketch of potential phases formed for differently ordered polymer:fullerene donor–acceptor multibulk heterojunctions.

In fact, it has been shown that the initially presented hypothesis is insufficient to describe the full complexity of the organic multiheterojunction. Indeed it is required to differentiate between a neat amorphous polymer (α) and a fullerene intercalated or intimately mixed amorphous polymer (*αβ*) fraction. Furthermore, even though it is not very probable to observe a direct interface between crystalline donor and acceptor in case of polymer‐based organic bulk heterojunctions, a charge transfer may possibly traverse (tunnel through) a thin interfacial layer of amorphously intermixed materials. Hence, the revisited model for the due to order varied interface between donor polymer and acceptor fullerene has to include in total six different CT‐transition energies, arising from contacts between all possible domains that can be formed within a bulk heterojunction. The corresponding sketch of involved domains and interfaces is depicted in **Figure** **8**.

It has been demonstrated, that variations in the due to order (semicrystalline) and disorder (amorphous) molecular energy levels located at the heterojunction, could be directly probed spectroscopically. In fact, several other methods (e.g., cyclic voltammetry and ultraviolet photon spectroscopy) are currently applied to shed light into the question on how to quantify the energetic changes that occur due to changes in phase order within bulk heterojunctions.^41^ The use of luminescence spectroscopy appears to be a very elegant way with small requirements to the equipment, yielding potentially deep insights into the energetics present at (multiple) organic bulk heterojunctions. However, this observation was specifically possible due to the fact that CT of PCBM combined with members from the polymer family AnE‐PV generally exhibit high luminescence quantum yields,^85^ making the observation of several CT peaks simply more probable. Experimentally a low‐noise luminescence measurement system perfects the requirements to enable this observation. Another advantage that has been made use of in this study is the in depth investigated behavior of ternary blends composed of amorphous and semicrystalline representatives of one and the same polymer backbone.^33^, ^34^, ^72^, ^73^, ^84^ This specifically allowed us to tune the order within the polymer domains at will and thus to tune and define precisely the system properties and the interfaces with the electron acceptor, PCBM. In summary, lower limits for the order–disorder induced LUMO energy difference of PCBM amounts to ≈134 meV, which is within range reported by Jamieson et al. for the electron affinity difference between aggregated and nonaggregated PCBM.^65^ For our polymer, bearing the identical backbone but different side‐chain substitutions, the lower limit for the HOMO energy level split between ordered and disordered phases amounts to ≈205 meV for neat amorphous polymer phases, which is in part already reflected by the obtainable open‐circuit voltages from earlier studies,^85^ and to ≈274 meV for amorphous polymer intimately mixed with PCBM.

## 3 Conclusion

In summary, we have demonstrated the existence of several CT transitions within one single organic semiconductor bulk heterojunction. By systematic variation of the order within the conjugated polymer phase through application of mixtures of semicrystalline and amorphous representatives of one and the same polymer backbone within ternary blends with the fullerene derivative PCBM, the domain order at the heterojunction interface could be tuned from ordered–ordered, over ordered–disordered, up to disordered–disordered. In direct relation with the respective interfacial area, the strength of the corresponding CT transition varied systematically and could be therefore unambiguously assigned via electroluminescence characterization. Via photoluminescence also fullerene‐intercalated amorphous polymer phases could be detected. Thus it could be shown that the CT transition can indeed be used to probe the interfacial order at the heterojunction, allowing deep insights into the internal structure of the bulk heterojunction by simple means.

Finally, the information of the different CT transition peaks could be transferred into knowledge about lower limits for disorder‐induced molecular energy level changes, such as the HOMO and LUMO: due to the unambiguous assignment of certain CT transitions to specific interfaces, lower limits for the energy relaxation due to order, or in other words, a lower limit for the difference in HOMO and LUMO levels between certain amorphous and semicrystalline phase domains could be derived.

In conclusion, sensitive luminescence spectroscopy on a properly designed ternary bulk heterojunction, bearing polymers with high luminescence quantum yields and tunable order, revealed both: the existence of six different CT energy levels depending on the domain order at the heterojunction as well as quantitative information about energy relaxation within organic semiconductors due to the effect of ordering.

## 4 Experimental Section

Ternary polymer:polymer:PCBM blends were prepared from mixtures of semicrystalline AnE‐PV*ab* and amorphous AnE‐PV*ba* with concentrations running from 0% to 100% AnE‐PV*ba* in steps of 10%. The global polymer:PCBM weight ratio was held constant at 2:3. The synthesis of the polymers is described elsewhere.^85^ PCBM was used as received from supplier Nano‐C. Donors and acceptors were dissolved in a 1:1 blend of chloroform and chlorobenzene assigned to be the optimal mixture promoting phase separation in AnE‐PV:PCBM blends.^86^ The solution concentration was 0.4 wt% of polymer part. Thin films of polymer:PCBM blends were spin cast on glass substrates for photoluminescence spectroscopy. Solar cell device preparation for electroluminescence spectroscopy on glass involved etching part of the ITO‐layer for selective contacting of the back electrode, followed by spin coating of PEDOT:PSS (Clevios PH, Heraeus). Deposited PEDOT:PSS films were annealed at 170 °C for 15 min to release residual moisture and immediately transferred to a nitrogen filled glovebox. The top aluminium electrode was deposited by physical vapor deposition yielding an active area of 0.5 cm^2^. Thin film steady‐state PL spectra and solar cell EL spectra were recorded with an Avantes AvaSpec ULS‐2048 fiber spectrometer. PL excitation was applied with a laser diode emitting at 405 nm. EL was conducted at an injection current of 100 mA applied with a Keithley 2601 Source Measure Unit. For PL normalization and evaluation of optical band‐gap, thin film transmission, and reflection spectra were recorded with a Varian Cary 5000 spectrophotometer under VW condition^87^ and reassembled to the thin film absorption spectra. For reference, all photovoltaic parameters on this set of devices have been published in ref. 33.

## Supporting information

As a service to our authors and readers, this journal provides supporting information supplied by the authors. Such materials are peer reviewed and may be re‐organized for online delivery, but are not copy‐edited or typeset. Technical support issues arising from supporting information (other than missing files) should be addressed to the authors.

Supplementary
