# Supplementary material for: Revelation of Interfacial Energetics in Organic Multiheterojunctions
Source: Adv Sci (Weinh). 2016 Dec 1;4(4):1600331. doi: 10.1002/advs.201600331 (PMC5396163; doi:10.1002/advs.201600331)
Supplement: Supplementary file 1 — Supplementary [file ADVS-4-na-s001.pdf]

Copyright WILEY-VCH Verlag GmbH & Co. KGaA, 69469 Weinheim, Germany, 2016.

ADVANCED SCIENCE
Open Access

Supporting Information

for *Adv. Sci.,* DOI: 10.1002/advs.201600331

Revelation of Interfacial Energetics in Organic Multiheterojunctions

Christian Kästner, Koen Vandewal, Daniel Ayuk Mbi Egbe, and Harald Hoppe*

Supplementary Information

Revelation of interfacial energetics in organic multi-heterojunctions

Christian Kästner[1,§], Koen Vandewal[2], Daniel A. M. Egbe[3], Harald Hoppe[1,$,†]

1 *Institute of Physics, Technische Universität Ilmenau, Weimarer Str.32, 98693 Ilmenau, Germany*

2 *Institut für Angewandte Photophysik, Technische Universität Dresden, George-Bähr-Str. 1, 01069 Dresden, Germany*

3 *Linz Institute for Organic Solar Cells, Johannes Kepler University Linz, Altenbergerstr. 69, 4040 Linz, Austria*

Current addresses:

§ *Institute of Thermodynamics and Fluid Mechanics, Technische Universität Ilmenau, Am Helmholtzring 1, 98693 Ilmenau, Germany*

$ *Center for Energy and Environmental Chemistry Jena (CEEC Jena), Friedrich Schiller University Jena, Philosphenweg 7a, 07743 Jena, Germany*

† *Laboratory of Organic and Macromolecular Chemistry (IOMC), Friedrich Schiller University Jena, Humboldtstrasse 10, 07743 Jena, Germany*

Definitions:

semi-crystalline polymer: AnE-PV*ab*

amorphous polymer: AnE-PV*ba*

chemical structures:

AnE-PV*ab*

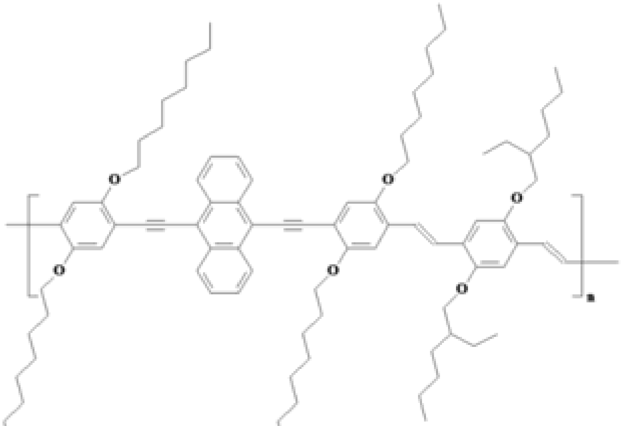

AnE-PV*ba*

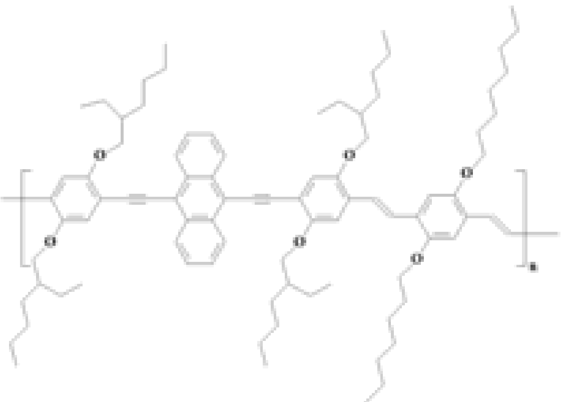

Voigt function:

$$V(x) = y_0 + A \cdot \frac{2\ln 2}{\pi^{3/2}} \frac{w_L}{w_G^2} \int_{-\infty}^{+\infty} \frac{e^{-t^2}}{\left(\sqrt{\ln 2}\,\frac{w_L}{w_G}\right)^2 + \left(\sqrt{4\ln 2}\,\frac{x - x_c}{w_G} - t\right)^2} dt$$

y_0: peak offset, A: amplitude, x_c: peak position, w_G: full width half maximum of Gauß function, w_L: full width half maximum of Lorentz function

TS1: Definition and calculation of energy levels

Initial values ($y_i \pm u_i$) obtained from spectroscopic analysis.

Mean value: $\bar{y} = \frac{1}{n}\sum_{i=1}^{n} y_i$

Total error: $u_y = \sqrt{\sum_{i=1}^{n} u_i^2}$

Final result: $y = \bar{y} \pm u_y$

PCBM-LUMO-splitting:

$$\Delta E_{PCBM}^{LUMO} = CT_1^{EL} - CT_3^{EL} = (127 \pm 36)\ meV$$

$$\Delta E_{PCBM}^{LUMO} = CT_1^{PL} - CT_3^{PL} = (141 \pm 20)\ meV$$

$$\overline{\Delta E_{PCBM}^{LUMO}} = (134 \pm 41)\ meV$$

Amorphous Polymer-HOMO-splitting (neat, *α*):

$$\Delta E_{\alpha}^{HOMO} = CT_1^{EL} - CT_2^{EL} = (205 \pm 34)\ meV$$

$$\overline{\Delta E_{\alpha}^{HOMO}} = (205 \pm 34)\ meV$$

Amorphous Polymer-HOMO-splitting (mixture, *αβ*):

$$\Delta E_{\alpha\beta}^{HOMO} = CT_1^{PL} - CT_2^{EL} = (275 \pm 21)\ meV$$

$$\overline{\Delta E_{\alpha\beta}^{HOMO}} = (275 \pm 21)\ meV$$

S1: Voigt-fits of electroluminescence spectra

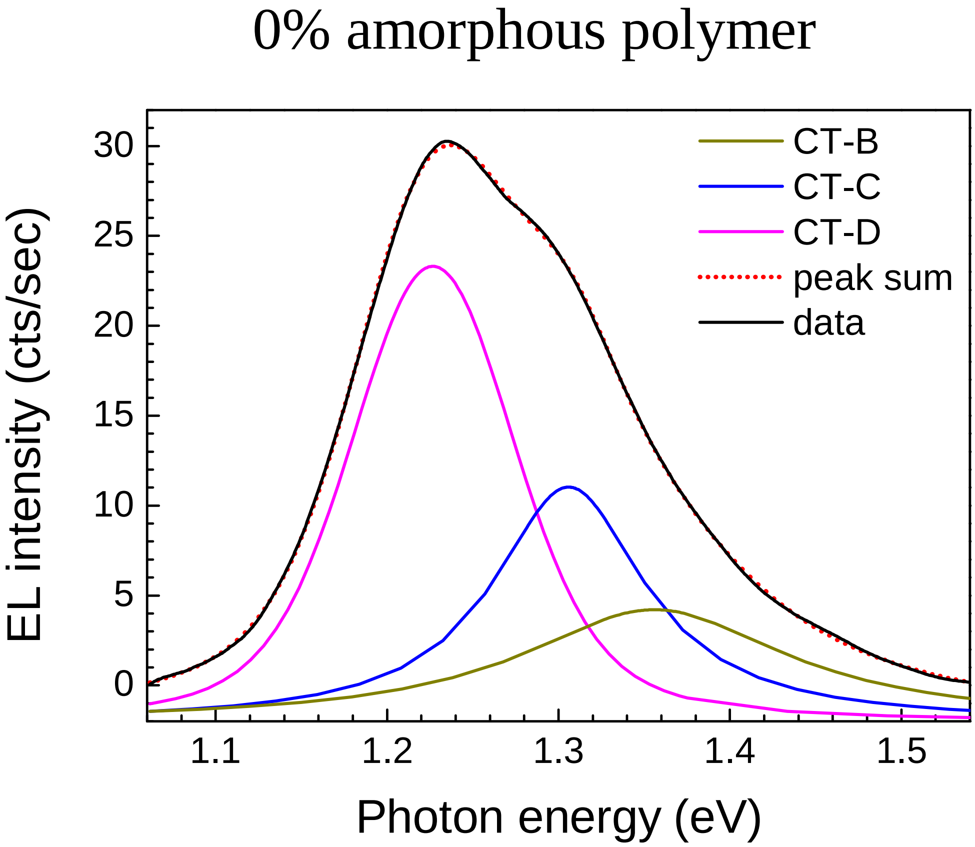

		Value	Standard Error
	x_C	-2.04261	0.06424
CT-B	A	1.35596	0.00386
	w_G	1.86497	0.17644
	w_L	6.00969E-9	44.42333
	x_C	0.18994	0.00473
CT-C	A	1.30592	3.94051E-4
	w_G	2.19548	0.14304
	w_L	3.28399E-9	14.24976
	x_C	0.10694	0.0024
CT-D	A	1.22668	2.26589E-4
	w_G	3.5592	0.02733
	w_L	0.09037	6.76888E-4
	x_C	0.04084	8.30905E-4

10% amorphous polymer

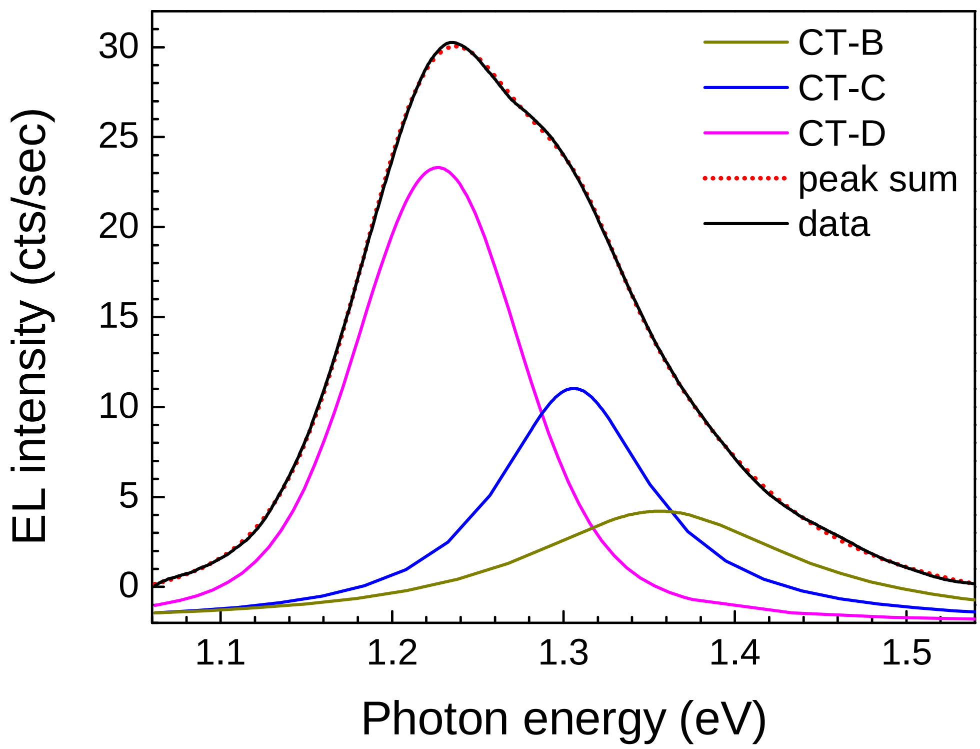

		Value	Standard Error
	y_0	-2.56738	0.19304
CT-B	x_C	1.36113	0.00693
	A	2.63752	0.39348
	w_G	0.13379	0.01278
	w_L	0.03604	0.00908
CT-C	x_C	1.30589	3.86861E-4
	A	4.68181	0.63823
	w_G	1.84761E-8	118.76166
	w_L	0.10436	0.00428
CT-D	x_C	1.23026	3.74133E-4
	A	4.72566	0.10768
	w_G	0.07792	0.00145
	w_L	0.04822	0.00229

20% amorphous polymer

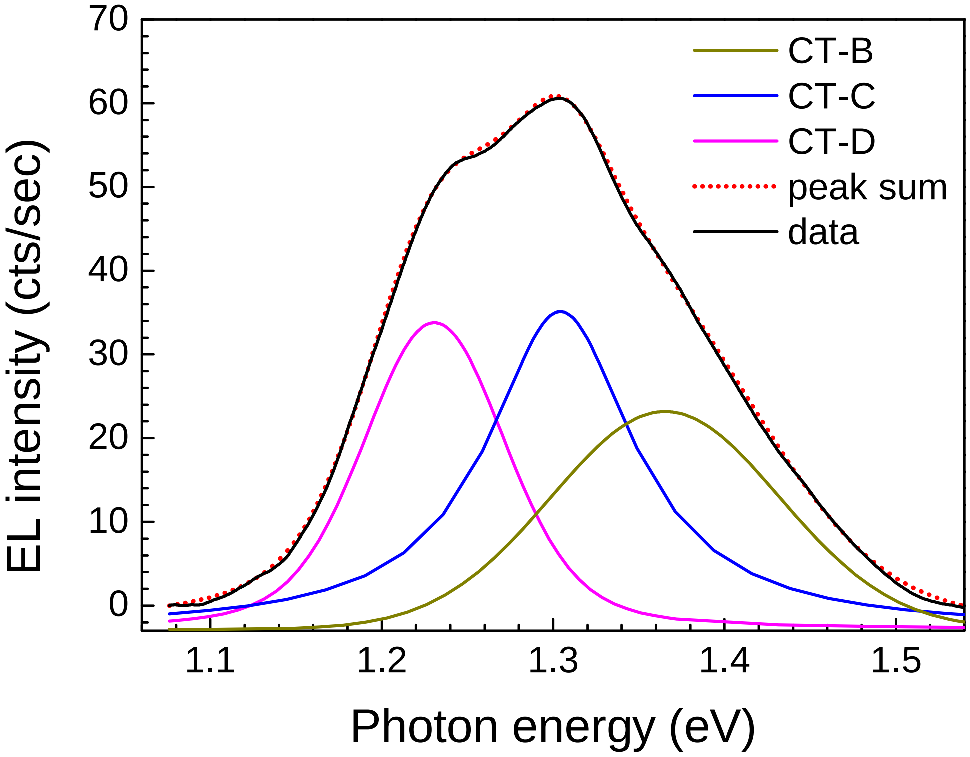

		Value	Standard Error
	y_0	-2.83125	0.10804
CT-B	x_C	1.36542	0.00231
	A	4.35033	0.23004
	w_G	0.15721	0.00312
	w_L	8.49495E-22	--
CT-C	x_C	1.30426	2.96287E-4
	A	6.13217	0.40266
	w_G	4.5696E-10	2.05956
	w_L	0.10291	0.00226
CT-D	x_C	1.23072	3.00674E-4
	A	4.31632	0.09461
	w_G	0.08212	0.00117
	w_L	0.02861	0.00226

30% amorphous polymer

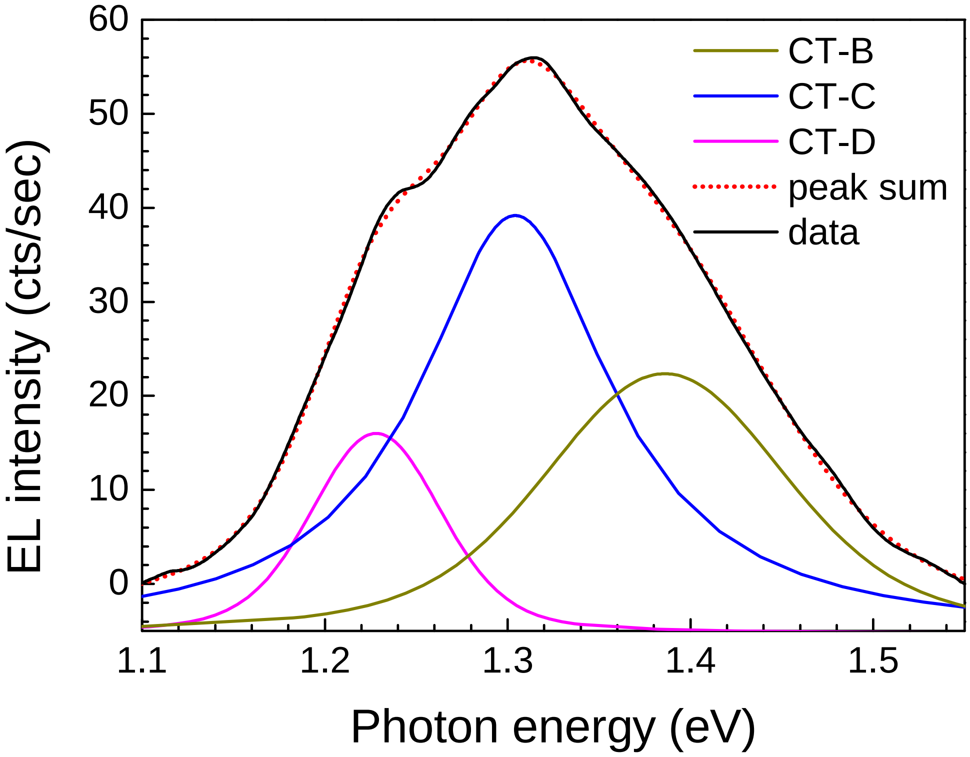

		Value	Standard Error
	y_0	-5.25353	0.49148
CT-B	x_C	1.386	0.0032
	A	5.43005	0.29759
	w_G	0.12011	0.01021
	w_L	0.06292	0.0107
CT-C	x_C	1.30416	5.53839E-4
	A	8.85699	0.85575
	w_G	3.11066E-9	16.95893
	w_L	0.12692	0.00491
CT-D	x_C	1.22809	8.20809E-4
	A	2.16654	0.21147
	w_G	0.06995	0.0026
	w_L	0.02568	0.00527

40% amorphous polymer

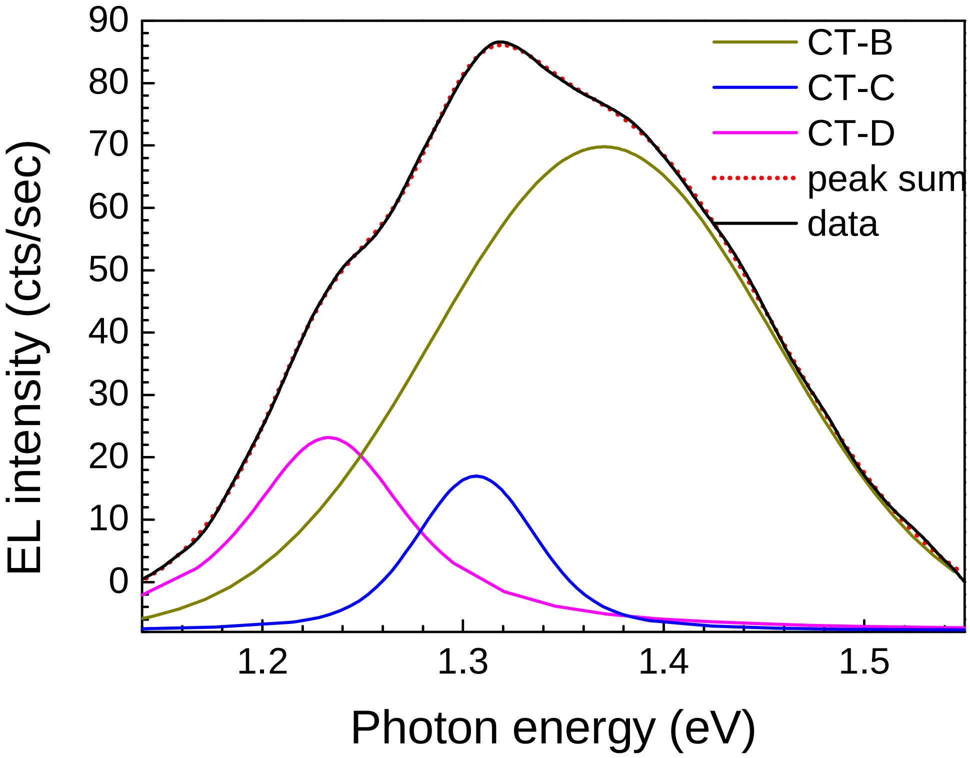

		Value	Standard Error
	y_0	-7.86198	0.21217
CT-B	x_C	1.37025	0.00165
	A	16.57172	0.43427
	w_G	0.20052	0.00226
	w_L	4.40345E-19	--
CT-C	x_C	1.30656	2.02716E-4
	A	2.20021	0.3989
	w_G	0.05485	0.00478
	w_L	0.02769	0.01179
CT-D	x_C	1.23293	2.62347E-4
	A	4.34718	0.07088
	w_G	0.03669	0.00302
	w_L	0.07931	0.00253

50% amorphous polymer

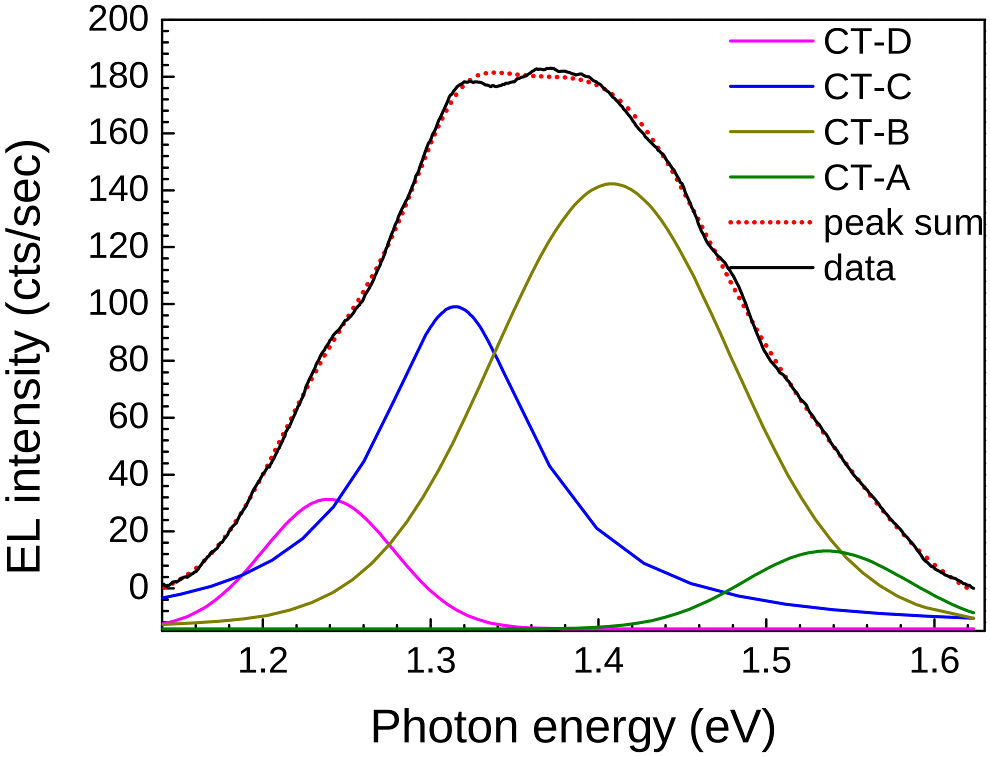

		Value	Standard Error
	y_0	-14.29955	5.56764
CT-A	x_C	1.53576	0.00855
	A	3.39089	2.06986
	w_G	0.11614	0.01897
	w_L	4.97071E-17	0.48938
CT-B	x_C	1.40796	0.00214
	A	29.37163	8.56439
	w_G	0.15867	0.02946
	w_L	0.0183	0.06516
CT-C	x_C	1.31468	8.55476E-4
	A	20.22168	4.5214
	w_G	4.52028E-8	438.35565
	w_L	0.11357	0.00865
CT-D	x_C	1.23919	0.00214
	A	4.44371	0.98404
	w_G	0.09155	0.0055
	w_L	2.71069E-223	24889.62643

60% amorphous polymer

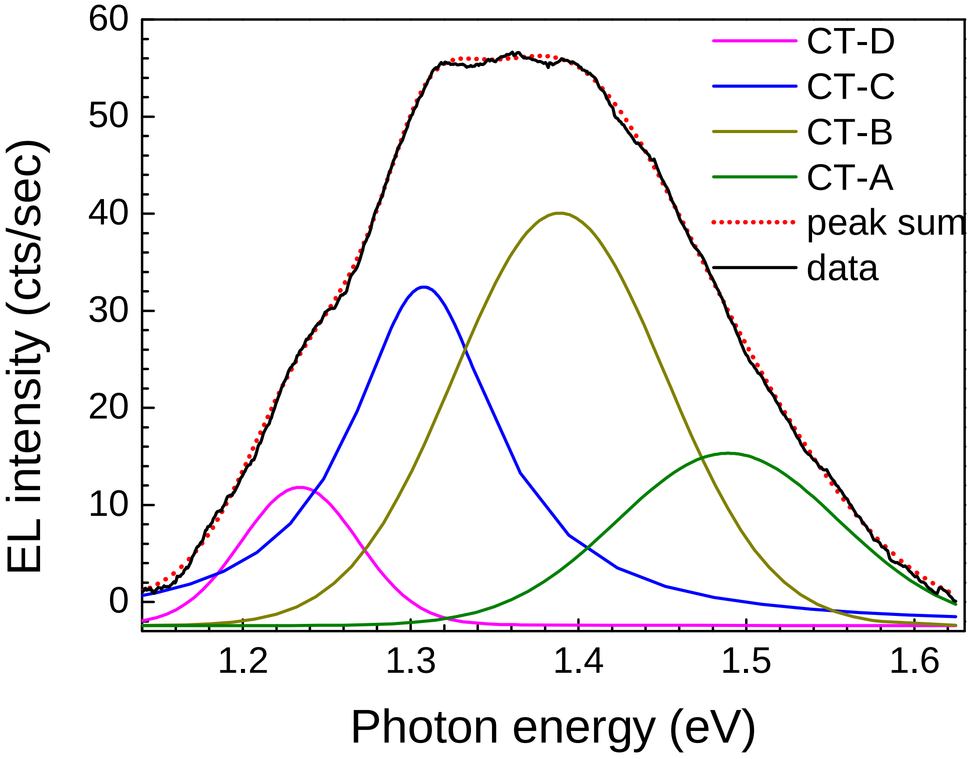

		Value	Standard Error
	y_0	-2.43035	0.56681
CT-A	x_C	1.4891	0.03946
	A	2.9514	2.42693
	w_G	0.15622	0.03375
	w_L	4.62835E-20	--
CT-B	x_C	1.3889	0.01111
	A	6.71097	3.04548
	w_G	0.1484	0.02122
	w_L	7.29244E-64	3.45612E8
CT-C	x_C	1.30782	5.62979E-4
	A	5.71275	1.28402
	w_G	1.04614E-9	9.23738
	w_L	0.10429	0.00671
CT-D	x_C	1.23408	9.56913E-4
	A	1.27781	0.19004
	w_G	0.08024	0.00519
	w_L	0.00428	0.01233

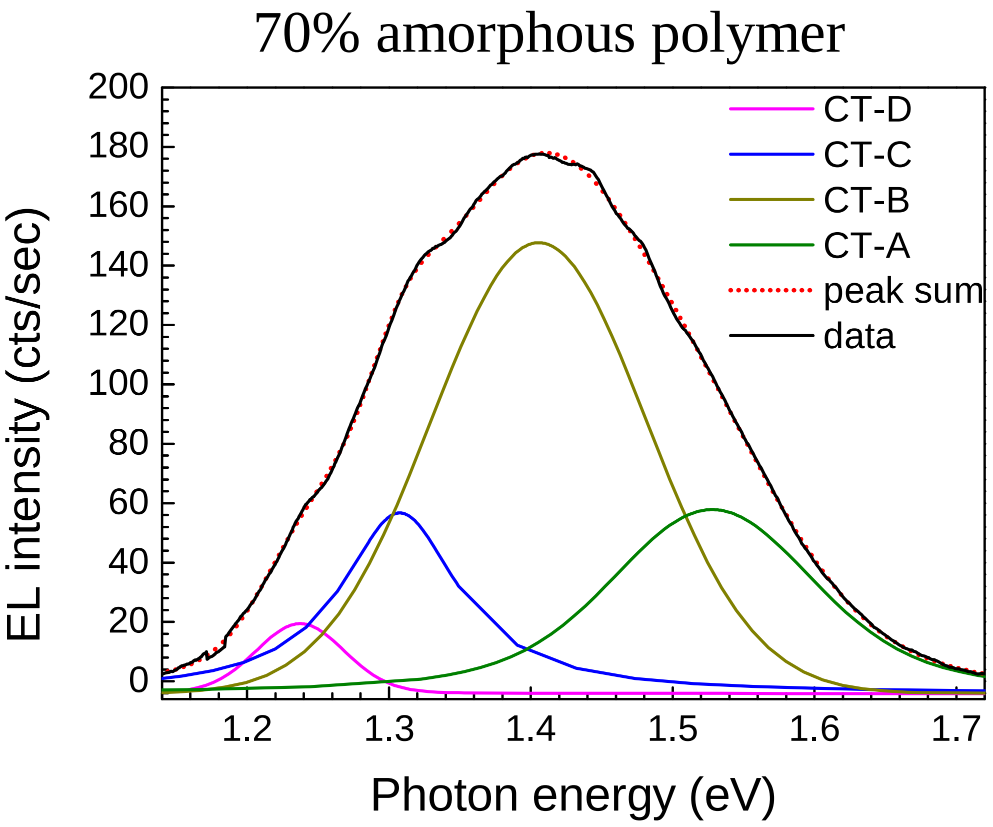

		Value	Standard Error
	y_0	-4.11552	0.47268
CT-A	x_C	1.52794	0.00568
	A	13.5771	1.57454
	w_G	0.12724	0.00788
	w_L	0.07571	0.00757
CT-B	x_C	1.40562	0.00229
	A	28.83695	1.96477
	w_G	0.17839	0.00655
	w_L	2.59572E-15	3.33334E-4
CT-C	x_C	1.30762	4.90725E-4
	A	9.59707	0.99075
	w_G	5.89942E-8	502.53694
	w_L	0.10044	0.00623
CT-D	x_C	1.23736	8.58502E-4
	A	1.92698	0.1711
	w_G	0.06986	0.00384
	w_L	0.00737	0.00745

80% amorphous polymer

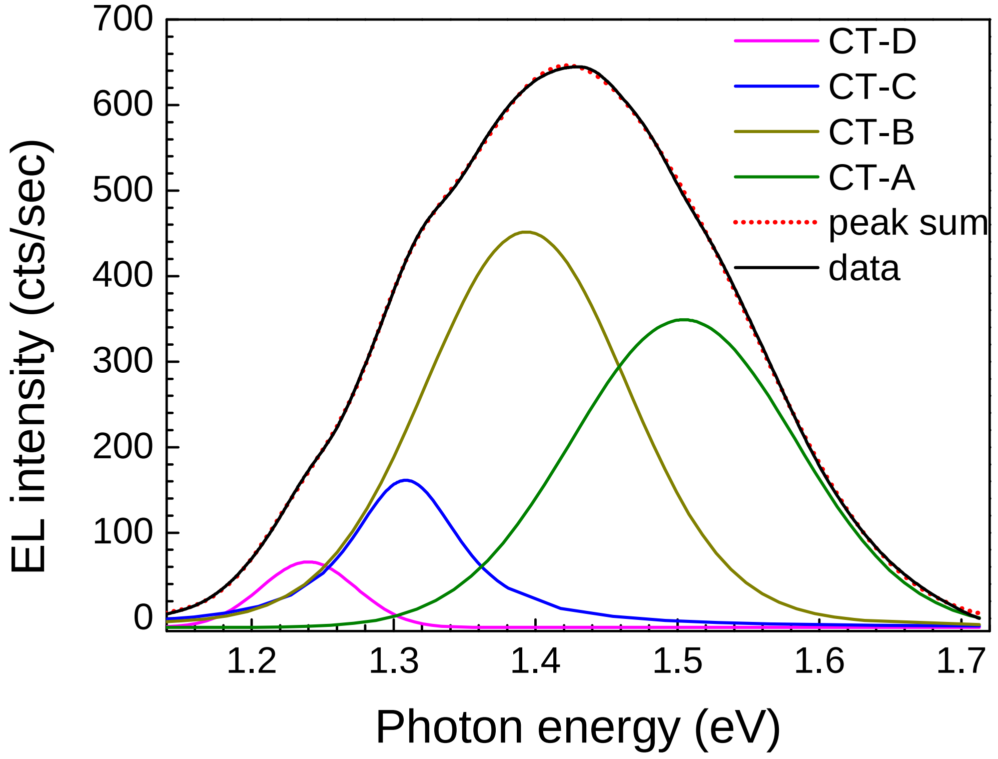

		Value	Standard Error
	y_0	-10.73334	0.85247
CT-A	x_C	1.5044	0.00939
	A	71.36593	12.49564
	w_G	0.18635	0.00875
	w_L	4.28713E-22	--
CT-B	x_C	1.3935	0.00359
	A	87.62432	19.45411
	w_G	0.15771	0.00571
	w_L	0.02112	0.01191
CT-C	x_C	1.30848	4.35475E-4
	A	23.40092	6.42076
	w_G	0.03546	0.00813
	w_L	0.077	0.01502
CT-D	x_C	1.23984	7.47976E-4
	A	6.45019	0.30247
	w_G	0.07906	0.00134
	w_L	1.80176E-14	3.43541E-4

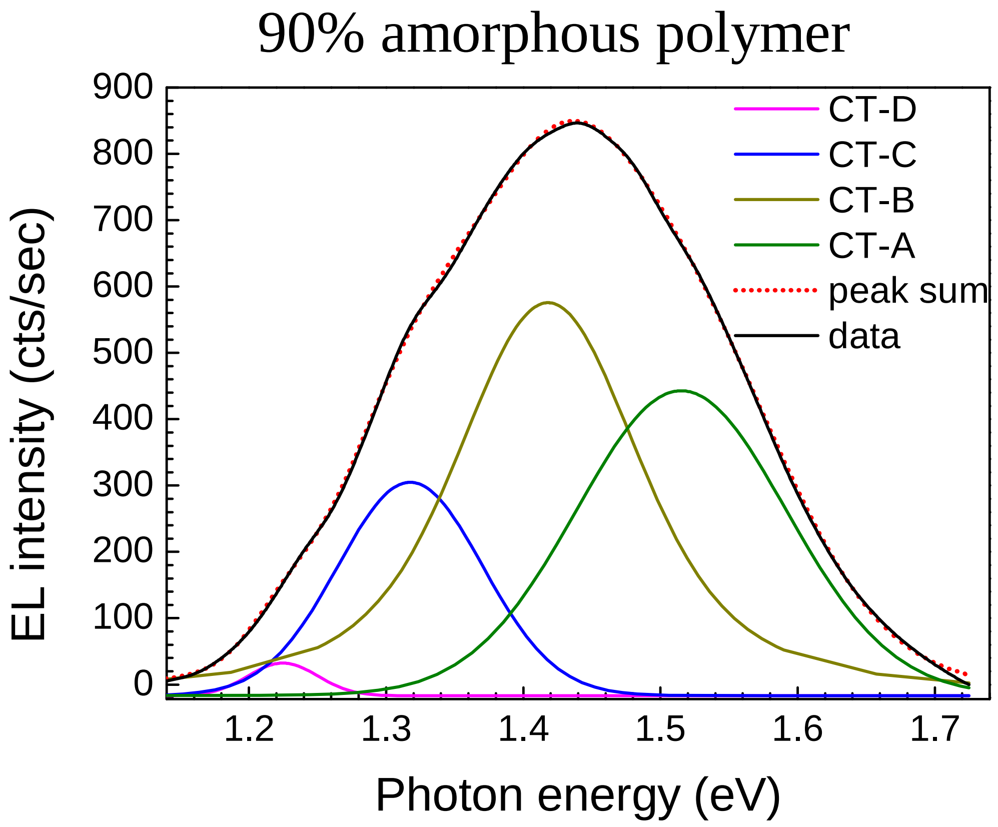

		Value	Standard Error
	y_0	-17.00998	7.43006
CT-A	x_C	1.51522	0.00694
	A	89.15641	21.4637
	w_G	0.17928	0.00824
	w_L	0.00303	0.01606
CT-B	x_C	1.41802	0.00337
	A	125.41899	48.46051
	w_G	0.10441	0.02253
	w_L	0.08804	0.04964
CT-C	x_C	1.31761	0.00451
	A	42.85527	16.68165
	w_G	0.12382	0.01343
	w_L	0.00133	0.03696
CT-D	x_C	1.22481	9.81152E-4
	A	3.13377	0.48751
	w_G	0.05963	0.00287
	w_L	3.01659E-38	32872.26446

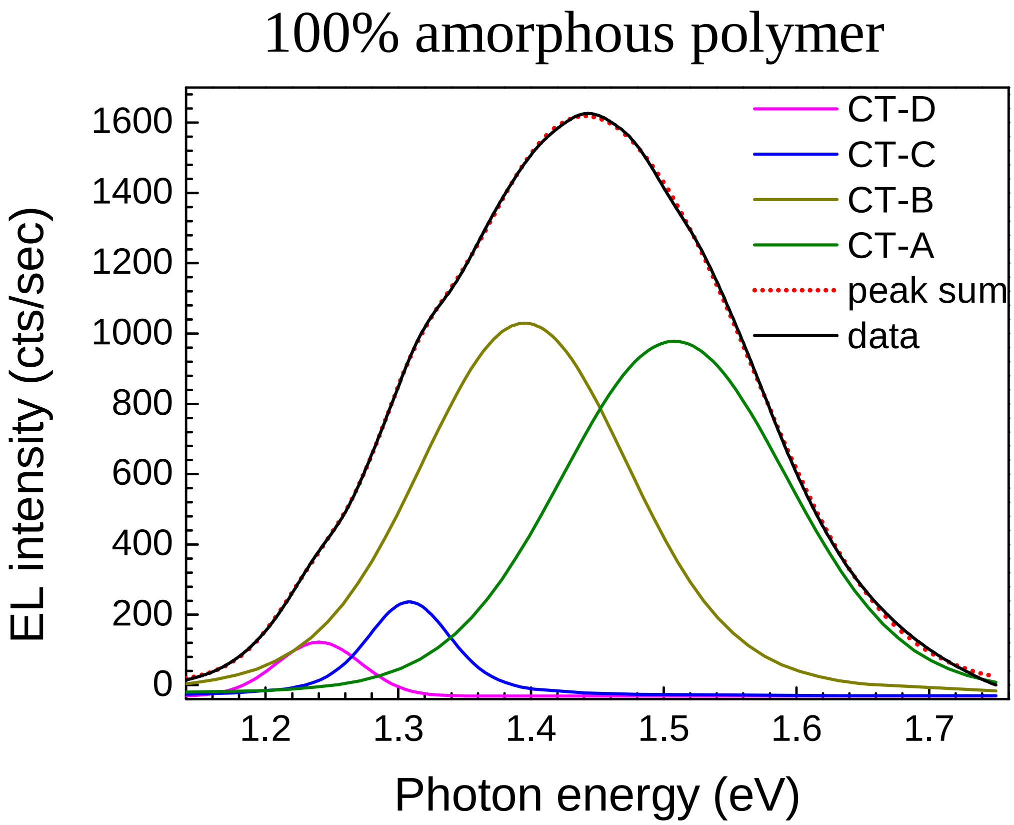

		Value	Standard Error
	y_0	-31.12681	3.42449
CT-A	x_C	1.50792	0.01295
	A	231.94586	50.33582
	w_G	0.18741	0.01106
	w_L	0.02943	0.00423
CT-B	x_C	1.39515	0.00654
	A	233.11996	65.86092
	w_G	0.16396	0.01757
	w_L	0.04311	0.00716
CT-C	x_C	1.30817	6.80578E-4
	A	26.13962	19.72987
	w_G	0.05558	0.01786
	w_L	0.03473	0.04737
CT-D	x_C	1.24012	0.00135
	A	12.25137	0.97564
	w_G	0.07524	0.00219
	w_L	2.97927E-14	7.80137E-4

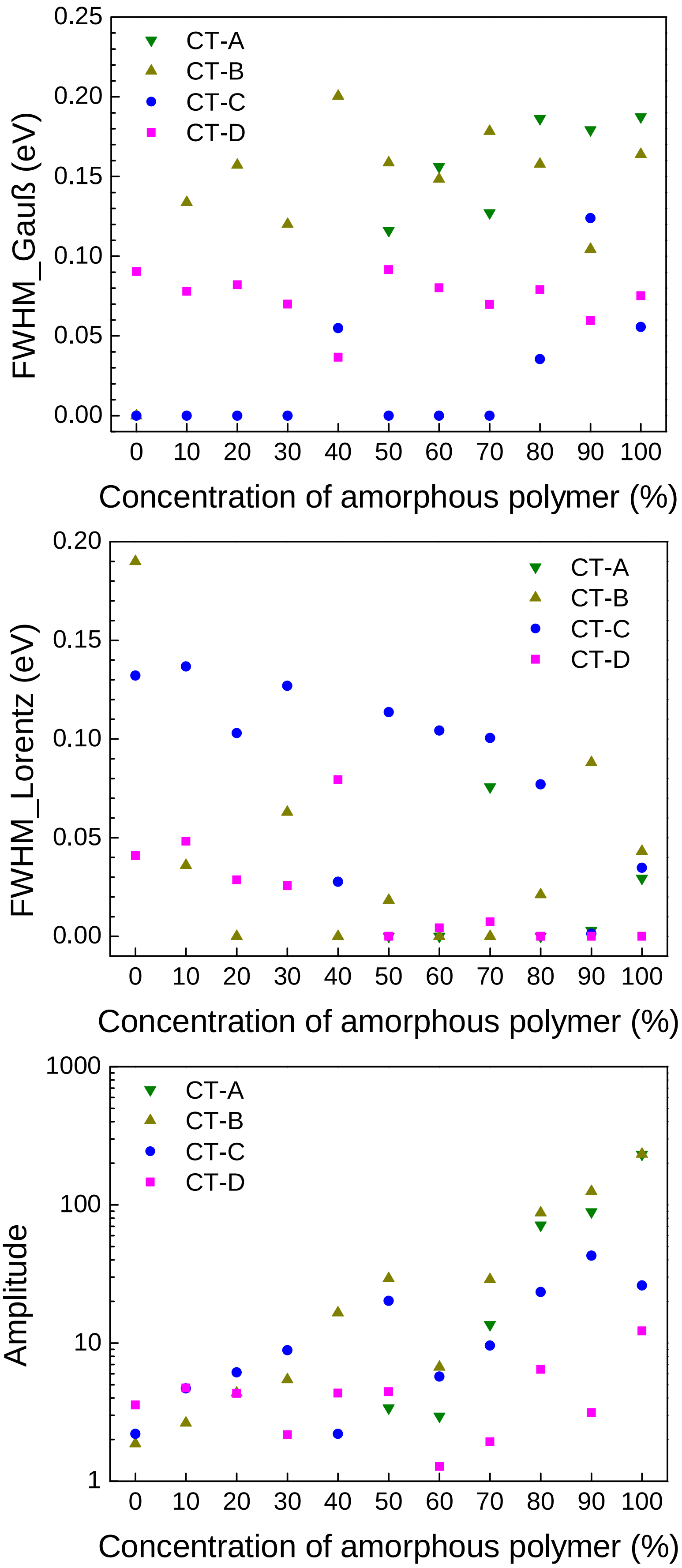

Charge Transfer	CT-A	CT-B	CT-C	CT-D
Interface	CT-1	CT-3	CT-2	CT-4

S2: Voigt-fits of photoluminescence spectra

Note: 0% and 10% of amorphous polymer did not provide sufficient signal strength for converging fits.

0% amorphous polymer

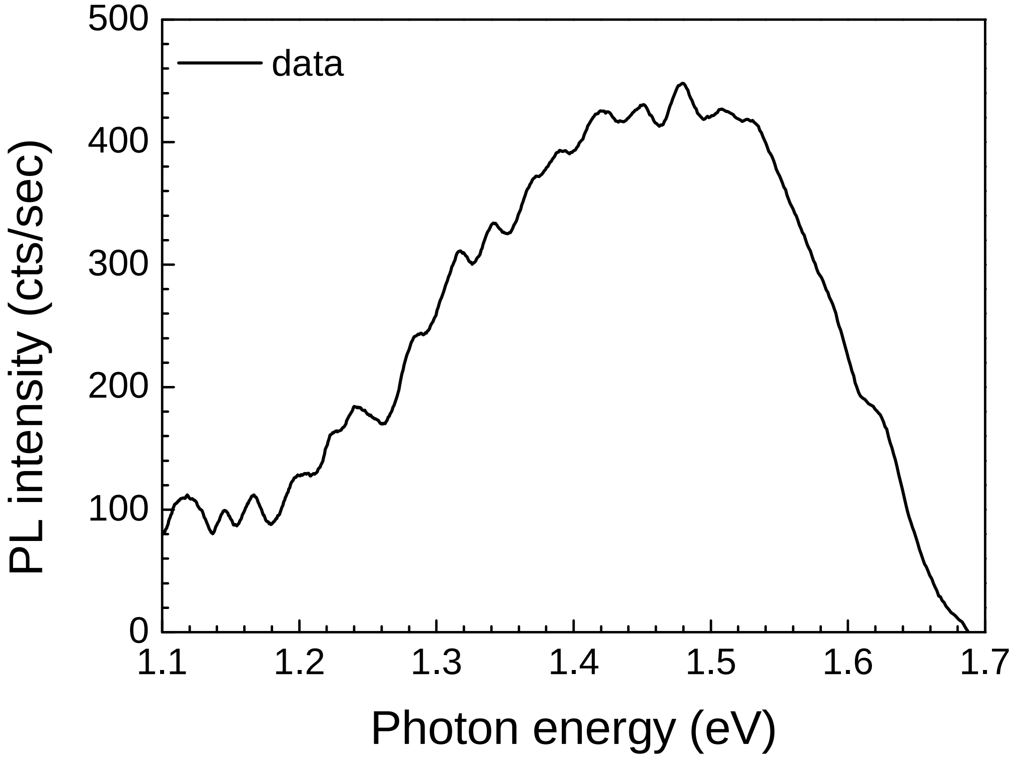

10% amorphous polymer

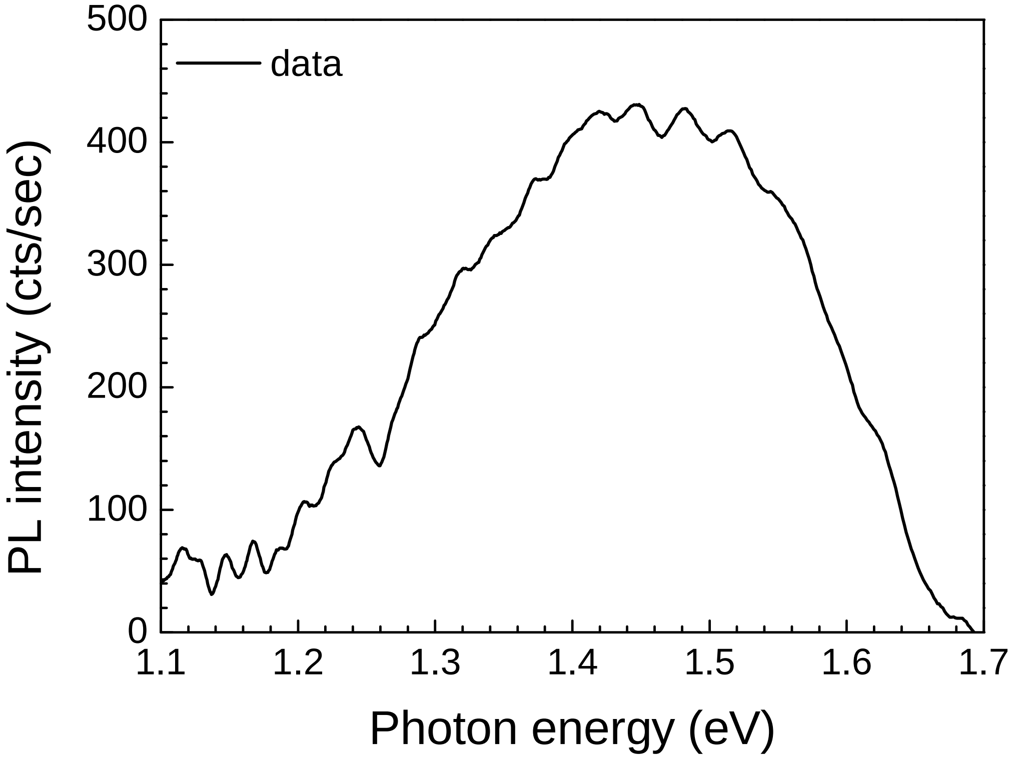

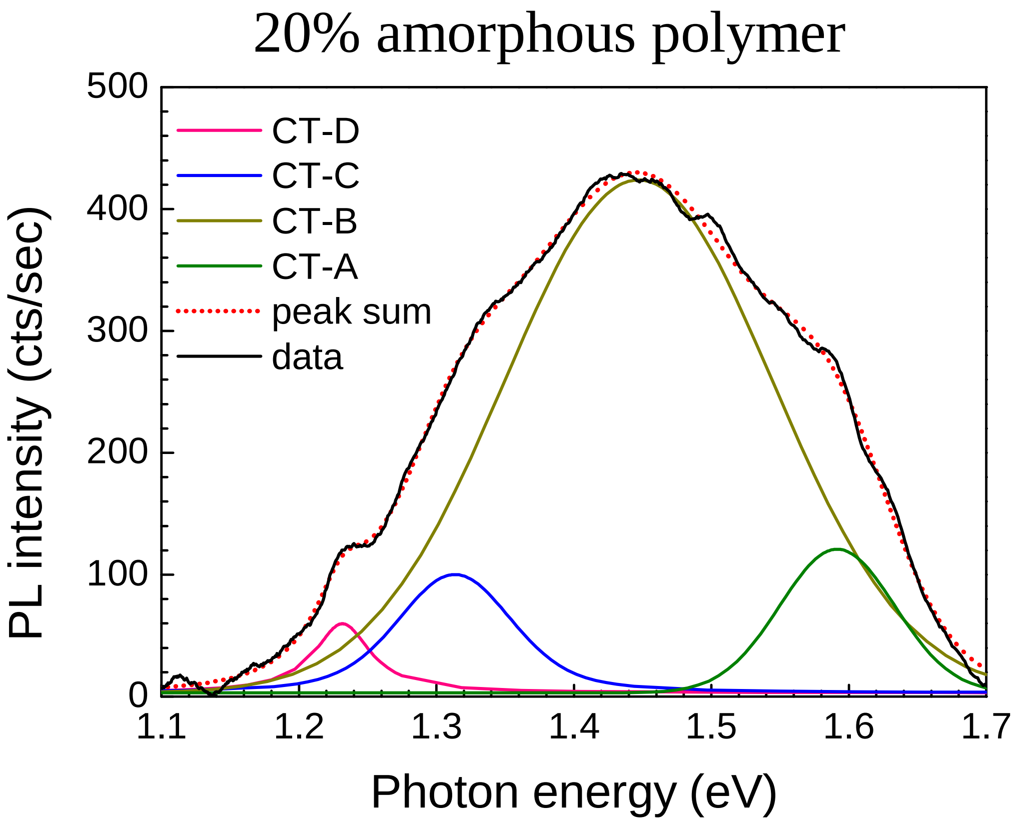

		Value	Standard Error
	y_0	3.06816	1.11042
CT-A	x_c	1.59157	0.000569368
	A	12.3454	0.85705
	w_G	0.09843	0.0027
	w_L	1.81E-20	--
CT-B	x_c	1.44703	1.29E-03
	A	103.0495	2.19029
	w_G	0.23006	0.00419
	w_L	3.97E-87	--
CT-C	x_c	1.31344	7.02E-04
	A	12.00364	2.61043
	W_G	7.73E-02	0.01067
	w_L	0.03803	0.0233
CT-D	x_c	1.23165	6.91E-04
	A	4.42908	0.52531
	w_G	3.29E-10	19.51286
	w_L	4.97E-02	0.0034

30% amorphous polymer

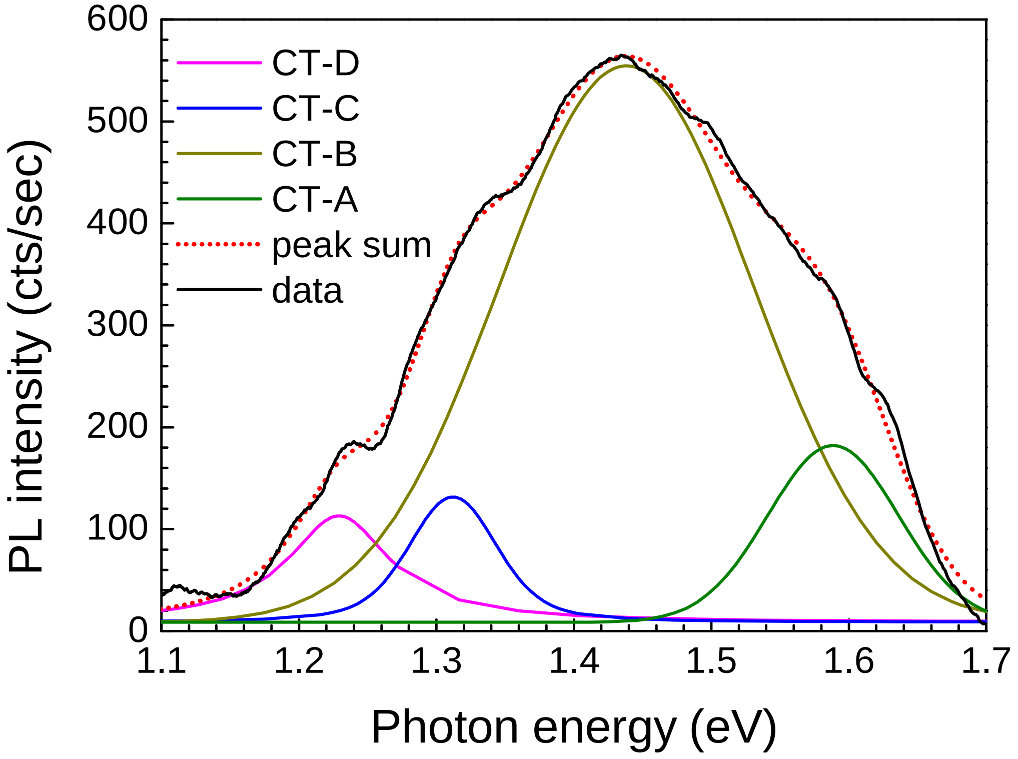

		Value	Standard Error
	y_0	3.06816	1.11042
CT-A	x_c	1.59157	0.000569368
	A	12.3454	0.85705
	w_G	0.09843	0.0027
	w_L	1.81E-20	--
CT-B	x_c	1.44703	1.29E-03
	A	103.0495	2.19029
	w_G	0.23006	0.00419
	w_L	3.97E-87	--
CT-C	x_c	1.31344	7.02E-04
	A	12.00364	2.61043
	W_G	7.73E-02	0.01067
	w_L	0.03803	0.0233
CT-D	x_c	1.23165	6.91E-04
	A	4.42908	0.52531
	w_G	3.29E-10	19.51286
	w_L	4.97E-02	0.0034

40% amorphous polymer

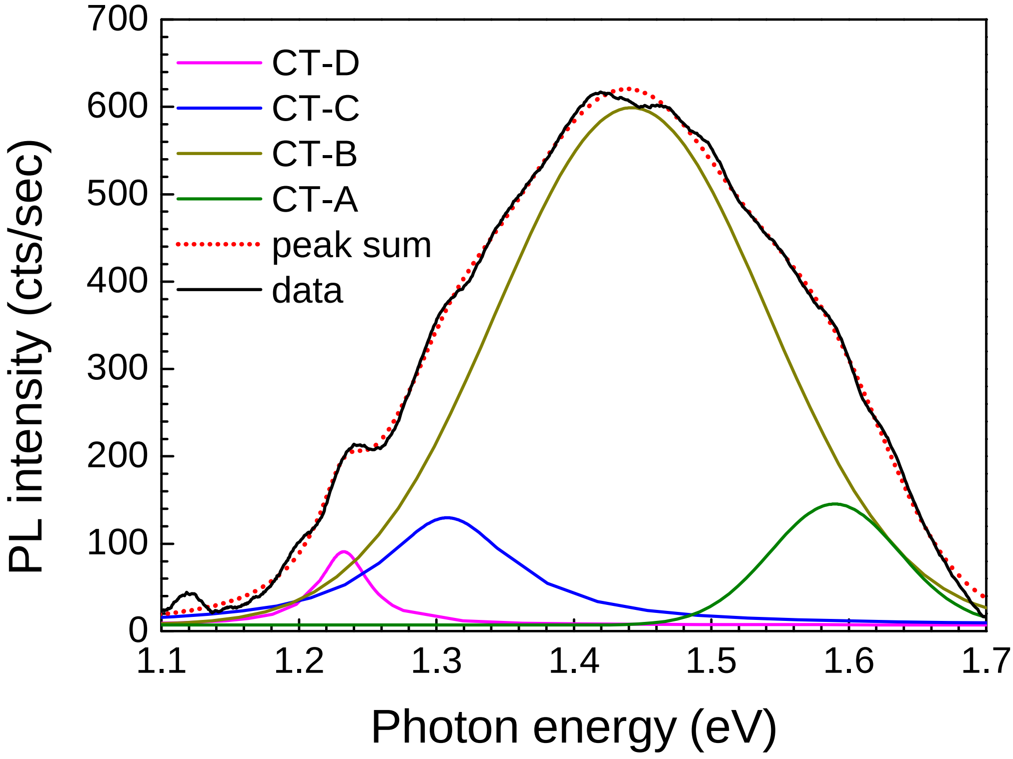

		Value	Standard Error
	y_0	6.95435	2.42299E-05
CT-A	x_c	1.58979	0.000400099
	A	16.23546	--
	w_G	0.11013	--
	w_L	7.25E-19	--
CT-B	x_c	1.44218	7.90E-04
	A	146.20226	2.61586
	w_G	0.23199	0.00301
	w_L	3.37E-46	366341000
CT-C	x_c	1.30789	1.11E-03
	A	22.32763	2.48652
	w_G	4.76E-26	3.15613E-15
	w_L	0.11576	0.00116
CT-D	x_c	1.23268	--
	A	5.71332	--
	w_G	5.145E-23	6.826E-12
	w_L	4.34E-02	--

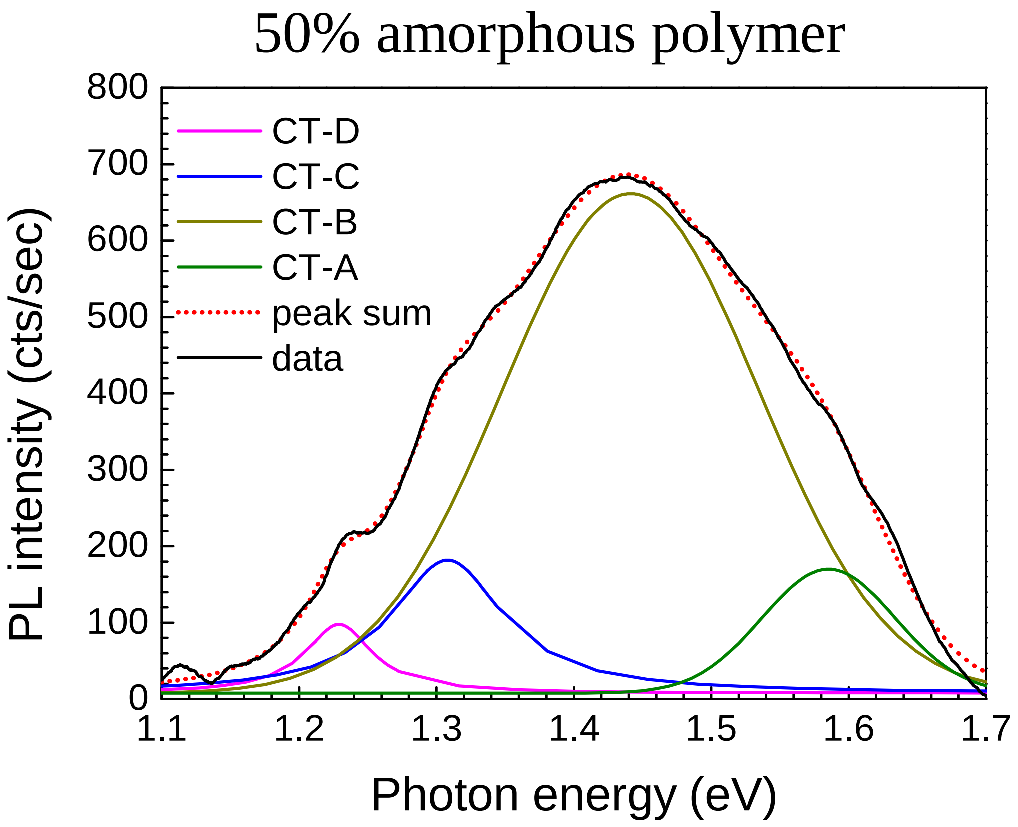

		Value	Standard Error
	y_0	7.51289	1.53519
CT-A	x_c	1.58534	0.00126
	A	19.72931	2.87514
	w_G	0.11419	0.00547
	w_L	1.13E-19	--
CT-B	x_c	1.44127	9.31E-04
	A	153.04294	5.33168
	w_G	0.2199	0.00661
	w_L	1.02E-15	1.77993E-05
CT-C	x_c	1.30806	7.05E-04
	A	27.01661	3.86773
	w_G	5.15E-09	147.60546
	w_L	0.09875	0.00603
CT-D	x_c	1.22906	7.78E-04
	A	8.39596	0.61034
	w_G	1.053E-14	0.000677947
	w_L	5.93E-02	0.00289

60% amorphous polymer

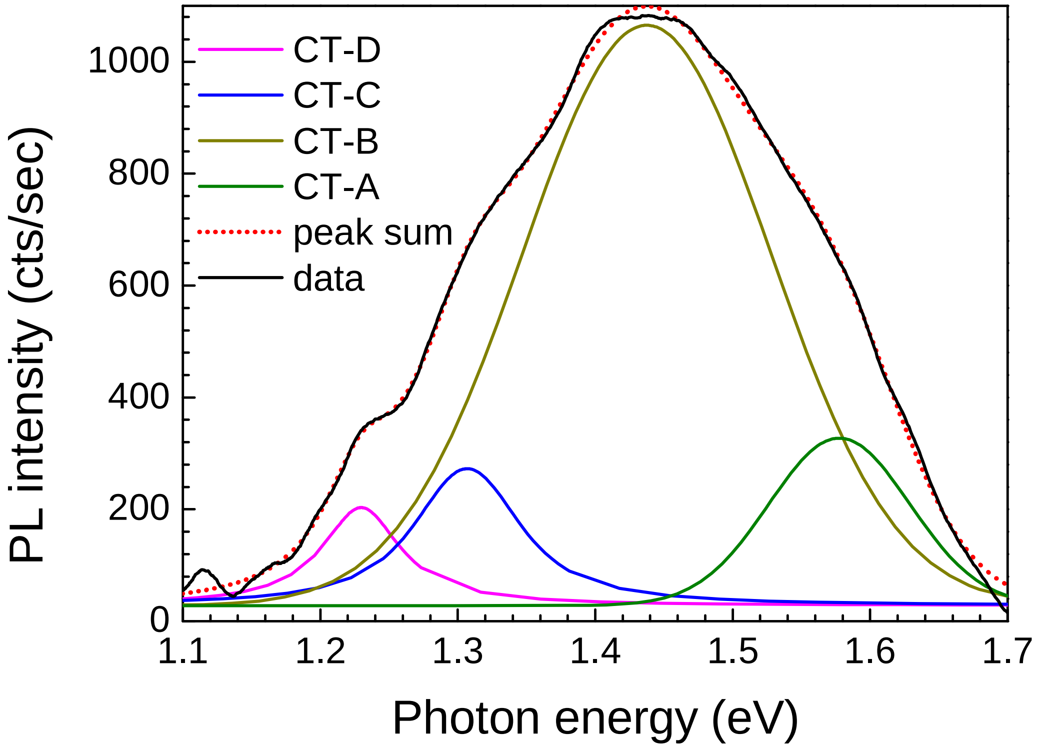

		Value	Standard Error
	y_0	28.06566	3.33625
CT-A	x_c	1.57754	0.00164
	A	38.43333	5.85265
	w_G	0.1207	0.00531
	w_L	7.68E-19	--
CT-B	x_c	1.43743	1.46E-03
	A	235.18954	10.32947
	w_G	0.21302	0.00772
	w_L	2.54E-62	--
CT-C	x_c	1.30688	6.82E-04
	A	32.99194	8.91922
	w_G	3.80E-02	0.01548
	w_L	0.07496	0.02158
CT-D	x_c	1.22974	7.76E-04
	A	19.06202	1.86019
	w_G	1.319E-09	57.0479
	w_L	6.93E-02	0.00319

70% amorphous polymer

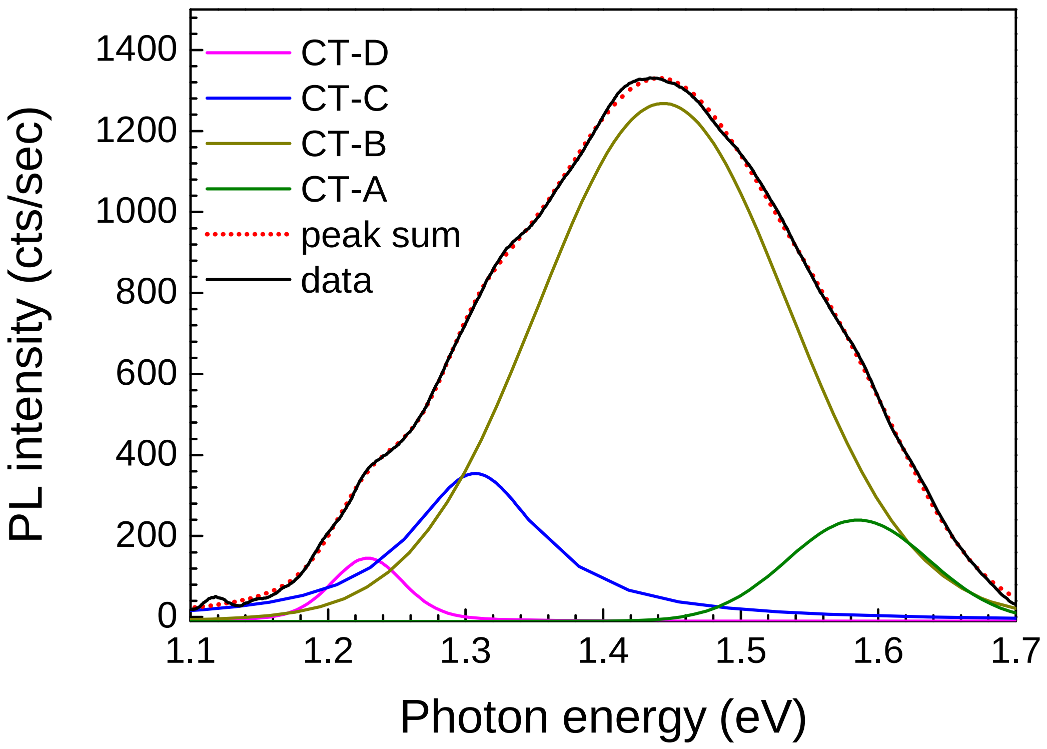

		Value	Standard Error
	y_0	-10.4245	6.56885
CT-A	x_c	1.58518	0.00149
	A	32.24438	3.90857
	w_G	0.12123	0.00435
	w_L	2.06E-16	0.00249
CT-B	x_c	1.44372	8.38E-04
	A	296.31297	8.12479
	w_G	0.21158	0.0067
	w_L	6.53E-03	0.00832
CT-C	x_c	1.30697	5.38E-04
	A	66.22967	9.49633
	w_G	2.88E-03	0.12565
	w_L	0.11552	0.01247
CT-D	x_c	1.22883	6.05E-04
	A	11.74517	2.58232
	w_G	0.05187	0.00699
	w_L	1.89E-02	0.01507

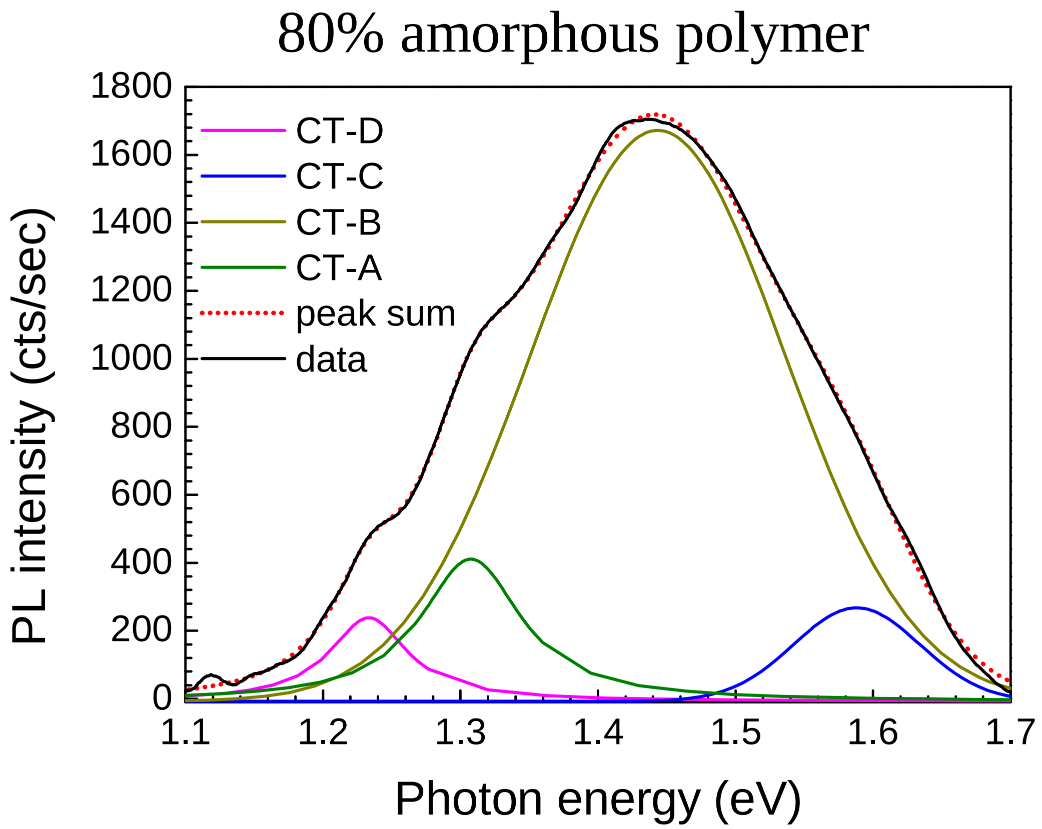

		Value	Standard Error
	y_0	-7.16263	2.56711
CT-A	x_c	1.58807	7.38E-04
	A	31.68722	2.76004
	w_G	1.08E-01	0.00333
	w_L	6.911E-19	--
CT-B	x_c	1.44345	5.10E-04
	A	390.17135	5.87144
	w_G	0.21833	0.00277
	w_L	4.08E-19	740971000
CT-C	x_c	1.30754	0.000349507
	A	57.07316	6.71789
	w_G	0.01731	0.01344
	w_L	8.45E-02	0.00934
CT-D	x_c	1.2333	4.87E-04
	A	26.479	1.502
	w_G	4.524E-08	1007.55503
	w_L	6.86E-02	0.00962

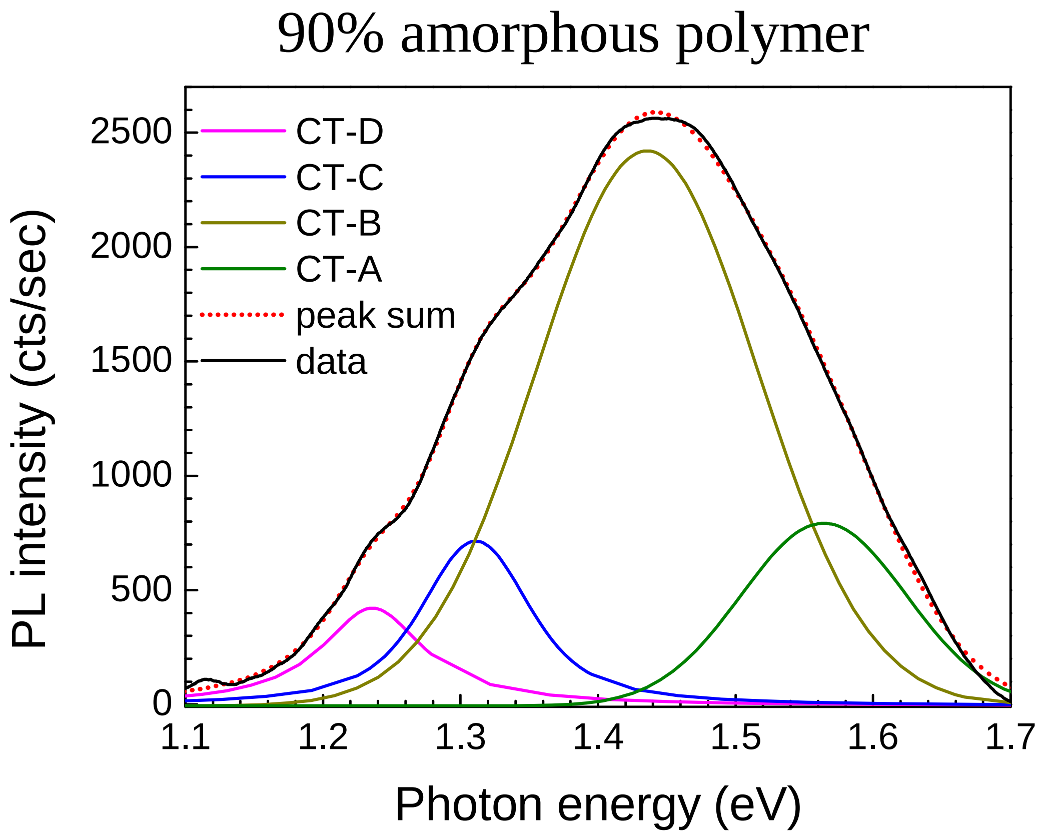

		Value	Standard Error
	y_0	-5.91152	10.33854
CT-A	x_c	1.56413	0.00634
	A	120.27363	33.32024
	w_G	0.14155	0.00722
	w_L	2.70E-19	--
CT-B	x_c	1.43561	2.44E-03
	A	488.87001	59.33013
	w_G	0.18927	0.01513
	w_L	1.47E-75	3441030000
CT-C	x_c	1.31111	6.99E-04
	A	93.79021	55.0039
	w_G	5.32E-02	0.02114
	w_L	0.06229	0.04539
CT-D	x_c	1.23592	1.40E-03
	A	61.0051	16.83625
	w_G	1.96E-06	171.89295
	w_L	9.09E-02	0.01573

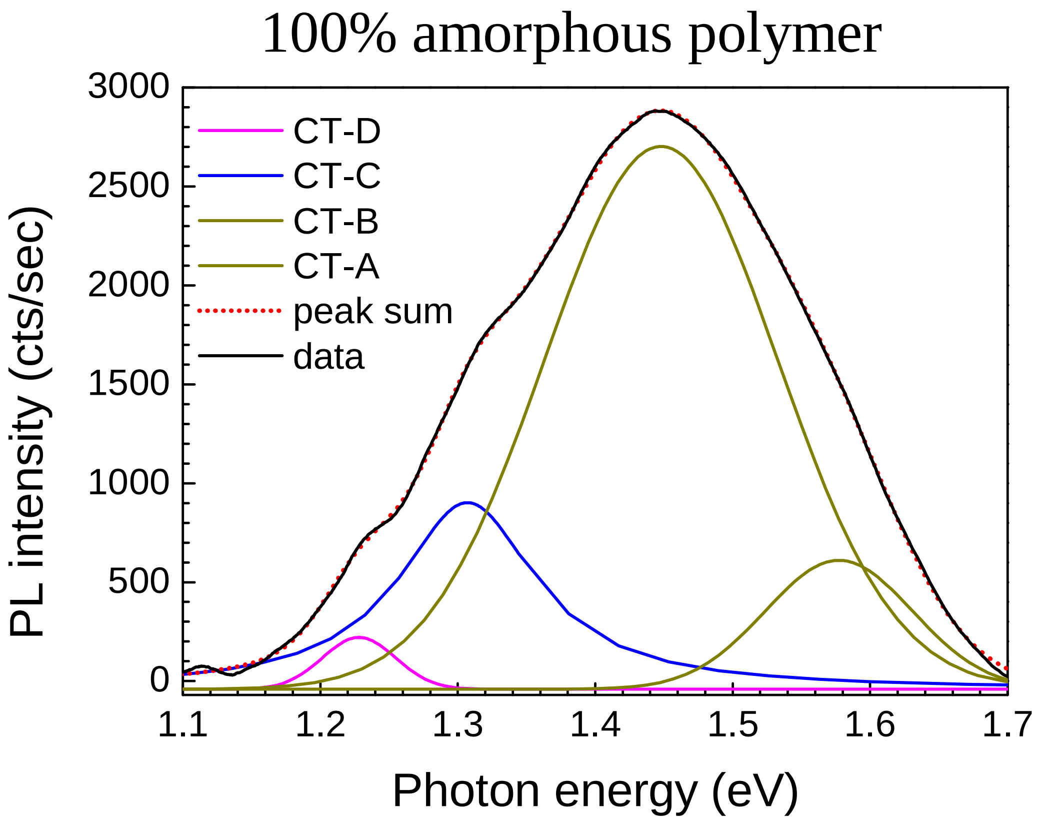

		Value	Standard Error
	y_0	-41.33851	3.98952
CT-A	x_c	1.57725	0.00149
	A	87.50601	8.98275
	w_G	0.12616	0.00328
	w_L	4.15E-19	--
CT-B	x_c	1.44794	9.59E-04
	A	583.83693	12.52879
	w_G	0.19994	0.00332
	w_L	1.57E-57	--
CT-C	x_c	1.30702	4.24E-04
	A	179.92622	6.84416
	w_G	6.52E-04	0.25756
	w_L	0.12145	0.00339
CT-D	x_c	1.22814	5.43E-04
	A	17.52346	0.71533
	w_G	0.06284	0.00129
	w_L	2.60E-57	--

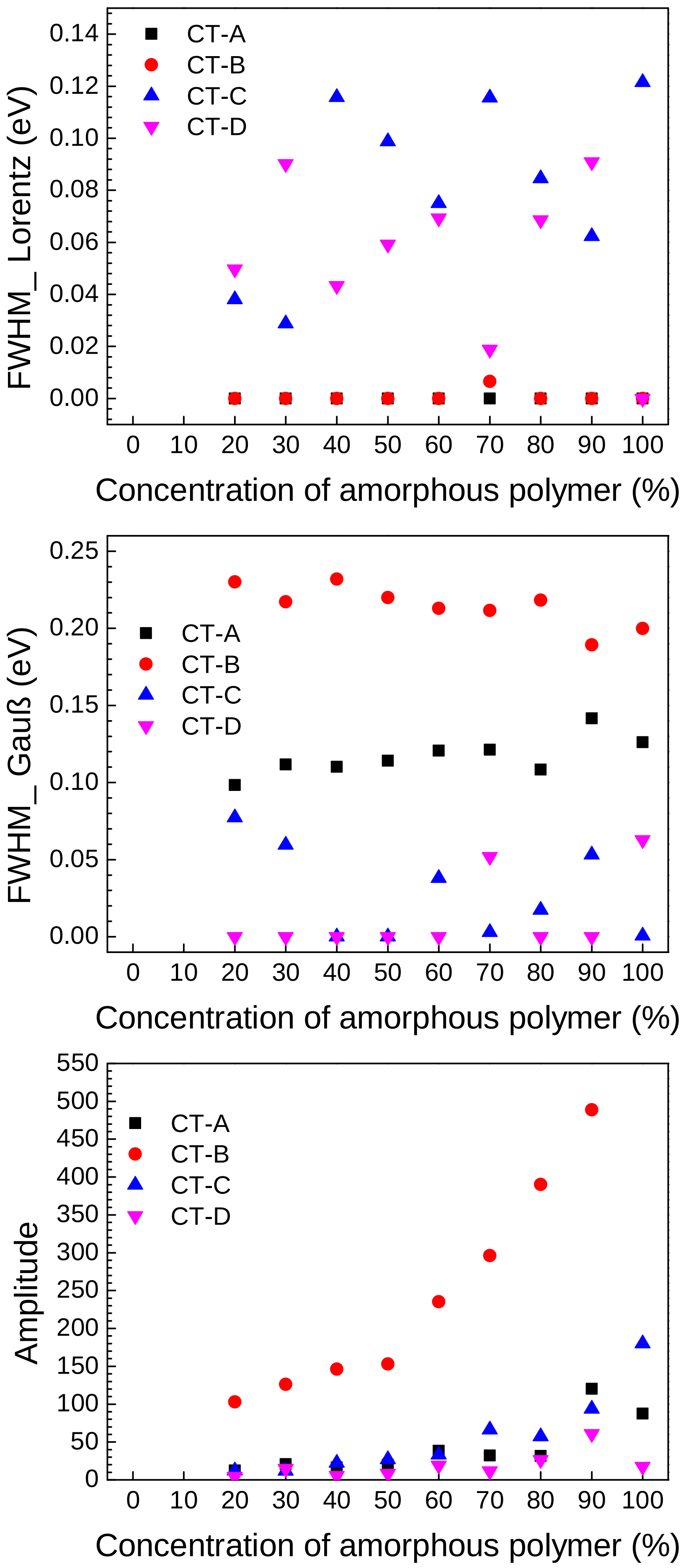
CT-A
CT-B
CT-C
CT-D
FWHM_ Lorentz (eV)
0.14
0.12
0.10
0.08
0.06
0.04
0.02
0.00
0
10
20
30
40
50
60
70
80
90
100
Concentration of amorphous polymer (%)
CT-A
CT-B
CT-C
CT-D
FWHM_ Gauß (eV)
0.25
0.20
0.15
0.10
0.05
0.00
0
10
20
30
40
50
60
70
80
90
100
Concentration of amorphous polymer (%)
CT-A
CT-B
CT-C
CT-D
Amplitude
550
500
450
400
350
300
250
200
150
100
50
0
0
10
20
30
40
50
60
70
80
90
100
Concentration of amorphous polymer (%)
